# A G protein–coupled receptor mediates neuropeptide-induced oocyte maturation in the jellyfish *Clytia*

**DOI:** 10.1371/journal.pbio.3000614

**Published:** 2020-03-03

**Authors:** Gonzalo Quiroga Artigas, Pascal Lapébie, Lucas Leclère, Philipp Bauknecht, Julie Uveira, Sandra Chevalier, Gáspár Jékely, Tsuyoshi Momose, Evelyn Houliston

**Affiliations:** 1 Sorbonne University, CNRS, Villefranche-sur-mer Developmental Biology Laboratory (LBDV), Villefranche-sur-mer, France; 2 Max Planck Institute for Developmental Biology, Tübingen, Germany; 3 Living Systems Institute, University of Exeter, Exeter, United Kingdom; Cornell University, UNITED STATES

## Abstract

The reproductive hormones that trigger oocyte meiotic maturation and release from the ovary vary greatly between animal species. Identification of receptors for these maturation-inducing hormones (MIHs) and understanding how they initiate the largely conserved maturation process remain important challenges. In hydrozoan cnidarians including the jellyfish *Clytia hemisphaerica*, MIH comprises neuropeptides released from somatic cells of the gonad. We identified the receptor (MIHR) for these MIH neuropeptides in *Clytia* using cell culture–based “deorphanization” of candidate oocyte-expressed G protein–coupled receptors (GPCRs). *MIHR* mutant jellyfish generated using CRISPR-Cas9 editing had severe defects in gamete development or in spawning both in males and females. Female gonads, or oocytes isolated from *MIHR* mutants, failed to respond to synthetic MIH. Treatment with the cAMP analogue Br-cAMP to mimic cAMP rise at maturation onset rescued meiotic maturation and spawning. Injection of inhibitory antibodies to the alpha subunit of the G_s_ heterodimeric protein (Gα_S_) into wild-type oocytes phenocopied the *MIHR* mutants. These results provide the molecular links between MIH stimulation and meiotic maturation initiation in hydrozoan oocytes. Molecular phylogeny grouped *Clytia* MIHR with a subset of bilaterian neuropeptide receptors, including neuropeptide Y, gonadotropin inhibitory hormone (GnIH), pyroglutamylated RFamide, and luqin, all upstream regulators of sexual reproduction. This identification and functional characterization of a cnidarian peptide GPCR advances our understanding of oocyte maturation initiation and sheds light on the evolution of neuropeptide-hormone systems.

## Abstract 2

A study of jellyfish oocytes identifies the receptor for Maturation-Inducing Hormone, the neuropeptide hormone that triggers oocyte maturation and spawning via GαS and cyclic AMP. This receptor defines a superfamily of hormone-receptor systems involved in regulating sexual reproduction across animal species.

## Introduction

Oocyte meiotic maturation is an essential process for animal sexual reproduction. It transforms the tetraploid, fully grown, ovarian oocyte into a haploid female gamete [1]. The core biochemical and cellular pathways operating within the oocyte during maturation are highly conserved between animals across the phylogenetic spectrum. Activation of the CyclinB-Cdk1 kinase complex assures meiotic progression from prophase I arrest into M phase, while parallel Mos-MAP kinase activation steers polar body formation as well as cytostatic arrest once maturation is complete [2,3]. In contrast, the upstream physiological processes vary widely, as does the molecular nature of the maturation-inducing hormones (MIHs) that act on the oocyte to trigger maturation [1,4]. This reflects clade-specific acquisition of endocrine tissues such as ovarian follicles, the corpus cardiacum/corpus allatum in insects, or the pituitary in vertebrates [5,6]. These tissues act downstream of other neuroendocrine sites such as the vertebrate hypothalamus to integrate environmental, behavioral, and physiological information in order to achieve optimal conditions for gamete development and release [7]. The complex evolutionary history of hormonal reproductive regulation has made it challenging to unravel the crucial regulatory events operating within the oocyte at the onset of maturation.

G protein–coupled receptors (GPCRs), the largest superfamily of integral transmembrane receptors [8], are good candidates to serve as MIH receptors (MIHRs). These 7-transmembrane domain proteins activate a variety of cytoplasmic signalling pathways via Gα and/or Gβγ subunits that become released from receptor-associated heterotrimeric Gαβγ protein upon ligand binding [9–11]. Members of the four main Gα subunits classes, Gα_s_, Gα_i_, Gα_q_, and Gα_12/13_, associate variously with members of the vast GPCR family. In vertebrates, constitutively active GPCRs are coupled to Gα_s_, which stimulates adenylate cyclase to maintain high cytoplasmic cyclic adenosine monophosphate (cAMP) concentrations in ovarian oocytes [12–14]. These high cAMP levels help hold the oocyte in an immature state, with the cell cycle arrested in meiotic prophase. The mechanisms by which MIHs override this arrest and initiate meiotic maturation vary between species and are not fully understood (see Discussion). In marked contrast, oocytes of many invertebrate species show a rise in cytoplasmic cAMP concentration upon MIH stimulation that is required for meiotic maturation [6]. In these species, GPCRs working through Gα_s_ are thus good candidates to trigger oocyte maturation, rather than to inhibit it as in vertebrates. The identification of such receptors could help to understand the origin of this diversity by providing an evolutionary perspective.

Here, we identify the MIHR in the hydrozoan jellyfish *Clytia hemisphaerica* as a GPCR likely working through Gα_s_, and determine its in vivo function. In *Clytia* and other hydrozoan species, MIH consists of PRPamide and related tetrapeptides that are released upon light stimulation from opsin-expressing gonad ectoderm cells, and act at the plasma membrane [15,16]. Neuropeptides such as these are of major importance in cnidarian biology, acting both as “neuroendocrine” mediators of physiological transitions, such as metamorphosis, as well as in fast neuromuscular transmission regulating swimming and feeding mediated by ligand-gated ion channels [17,18]. We uncovered an oocyte-expressed Class A GPCR in *Clytia* (MIHR) activated by MIH peptides, which is to our knowledge the first characterized cnidarian neuropeptide GPCR [19,20]. Antibody inhibition experiments further provided evidence that Gα_s_ links MIHR activation to cAMP production to trigger maturation [21,22]. CRISPR-Cas9 mediated mutation of the *MIHR* gene revealed an essential in vivo function of *MIHR* in initiating oocyte maturation. Phylogenetic analysis of the MIHR sequence identified an evolutionary link to a subset of bilaterian neuropeptide-hormone GPCR families, including several upstream regulators of reproduction. These results allow us to propose a new scenario for the evolution of hormonal signalling pathways regulating oocyte maturation.

## Results

### Selection of candidate MIH GPCRs

Amidated neuropeptides like *Clytia* MIH commonly signal through GPCRs, although other receptor types can also be used [23]. As the first step to select candidate MIH receptors, we compiled a comprehensive catalogue of *Clytia* GPCRs from a *Clytia* reference transcriptome covering all life cycle stages. Our bioinformatics pipelines first retrieved all sequences predicted to code for 7 transmembrane domain (7TM) proteins and bearing GPCR-related Pfam database tags. An initial list of 761 sequences was then assigned by Pfam to the three main GPCR classes: A (rhodopsin-like), B (secretin-like), and C (metabotropic glutamate-like) [8,24] or to an “other” category (which included, for instance, sweet-taste receptors and cAMP-like receptors). The final dataset of 536 class-sorted putative *Clytia* GPCRs obtained after removal of duplicates (S1 Text) may be a slight overestimate due to some incorrectly identified or incomplete sequences. We focused on the 377 class-A GPCRs, because most neuropeptide GPCRs belong to this class [8,25].

**Fig 1:**
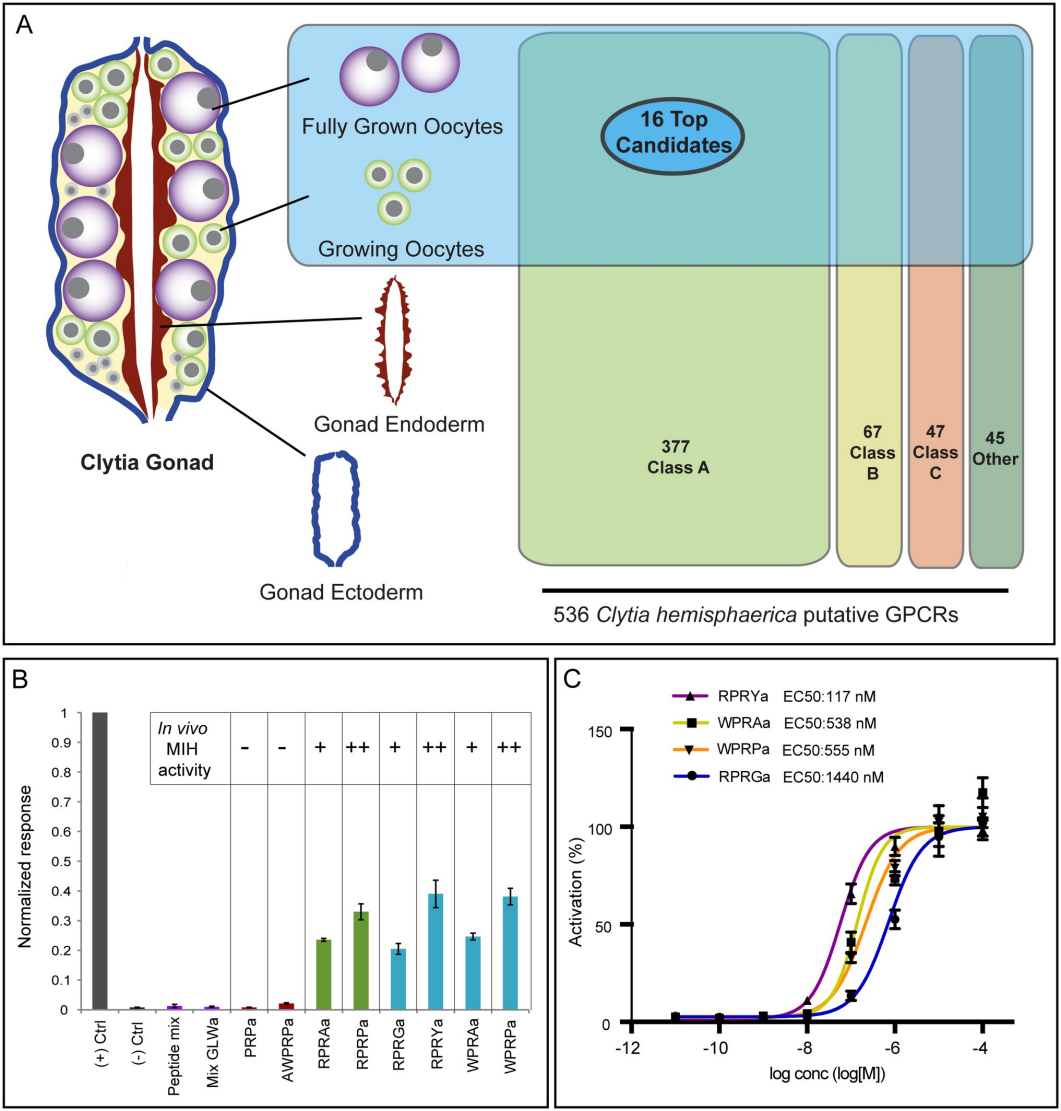
Identification of the *Clytia* MIHR. (A) Diagram of a *Clytia* gonad showing the different tissues used for RNA-seq and GPCR expression comparisons. A total of 536 putative GPCRs identified in *Clytia* mixed-stages transcriptome were assigned to the three main GPCR classes (A, B, C) or “other” based on Pfam signatures. The 16 top candidate MIH GPCRs were selected based on oocyte enrichment, relatedness to known bilaterian class-A neuropeptide GPCRs, and Pfam information. (B) Luminescence response of CHO-K1 cells expressing the putative *Clytia* MIH GPCR treated with neuropeptide mixes lacking MIH activity (purple bars), MIH tetrapeptides identified from *C*. *hemisphaerica* (blue bars) or *Cladonema radiatum* (green bars), or related penta- and tripeptides previously shown to be ineffective in triggering oocyte maturation in vivo (red bars) [15]. Empty pcDNA3.1 vector was used as negative control and *Platynereis* FLamide and its receptor as a positive control (gray bars). Peptide concentrations all 1 μM. Absolute units of luminescence were normalized using the positive control; data are shown as mean ± standard error of the mean (*n* = 3). Full datasets are available in S1 Data. MIH tetrapeptides were selectively able to activate the *Clytia* GPCR, the responses closely matching the in vivo MIH activity of each peptide tested on *Clytia* oocytes as indicated (summarized results from [15]). (C) Dose–response curves of *Clytia* MIHR challenged with four variant *Clytia* MIH tetrapeptides. One of three independent experiments with equivalent results is shown. Luminescence values were normalized relative to the maximum of fitted dose–response curves and are shown as mean ± standard error of the mean (*n* = 3). Half maximal effective concentration (EC_50_) values were calculated as means of 3 independent experiments. Full datasets from three independent experiments are available in S2 Data. CHO-K1, Chinese Hamster ovary K1; GPCR, G protein–coupled receptor; MIH, maturation-inducing hormone; MIHR, MIH receptor; RNA-seq, RNA sequencing.

An important criterion for the selection of candidate MIH receptors from the class-A GPCR list was enrichment in oocytes (Fig 1A). We mapped Illumina HiSeq mRNA reads previously obtained from *Clytia* gonad tissues [16] and life cycle stage [26] against all putative GPCR sequences. Profile clustering revealed three groups of sequences with oocyte-enriched expression (S1 Fig). We further narrowed down the number of potential MIHRs from these expression groups to 96 sequences, taking into account also Pfam indicators and sequence similarity with a set of bilaterian GPCRs [25]. Finally we compiled a shortlist of 16 candidates for functional testing using high expression level in oocytes as the final selection criterion (Fig 1A; S2 Text).

### Identification of the *Clytia* MIH receptor by GPCR deorphanization assay

We used a cell culture–based GPCR deorphanization assay to identify the MIH receptor from our candidate shortlist. cDNAs for each candidate were transfected into Chinese Hamster ovary K1 (CHO-K1) cells along with the promiscuous Gα protein Gα-16, and an aequorin-GFP luminescence reporter that measures Ca^2+^ mobilization downstream of this heterologous Gα protein [27,28]. We first screened the 16 candidate *Clytia* GPCRs against a mixture of 33 synthetic amidated peptides (including MIHs) predicted to be generated from previously identified putative *Clytia* neuropeptide precursors [15] and one additional one identified from transcriptome data. Given our imperfect knowledge of pro-peptide processing in cnidarians, some of the synthetic peptides may not correspond to endogenous peptides [29,30]. Only one of the 16 GPCRs was activated by this peptide mixture. This receptor responded well to all 4 synthetic *Clytia* MIH tetrapeptides, as well as to related *Cladonema* MIH peptides that can also activate *Clytia* oocyte maturation, but not to other *Clytia* peptide mixtures or poorly active MIH penta-/tripeptides. We termed this *Clytia* GPCR the Maturation Inducing Hormone Receptor (MIHR). The activity of individual peptides at 1 μM to stimulate the MIHR closely matched their in vivo ability to induce oocyte maturation [15] (Fig 1B).

The *Clytia* MIH amidated peptides are produced from two precursor genes *Che-pp4* (generating multiple copies of WPRAamide, WPRYamide, and WPRPamide) and *Che-pp1* (multiple copies of WPRPamide and RPRGamide) [15]. Their predicted structural similarity suggests they bind the same site on the receptor with different affinities. Dose–response curves for each of the 4 *Clytia* MIH neuropeptides, generated from three independent experiments, showed half maximal effective concentration (EC_50_) values in the high-nanomolar or low-micromolar range for all 4 MIHs, with RPRYamide showing the highest activity and RPRGamide the lowest (Fig 1C). To our knowledge, *Clytia* MIH and MIHR are the first neuropeptide ligand-GPCR pair demonstrated in a cnidarian.

### Phenotypes of *MIHR* mutant jellyfish

**Fig 2:**
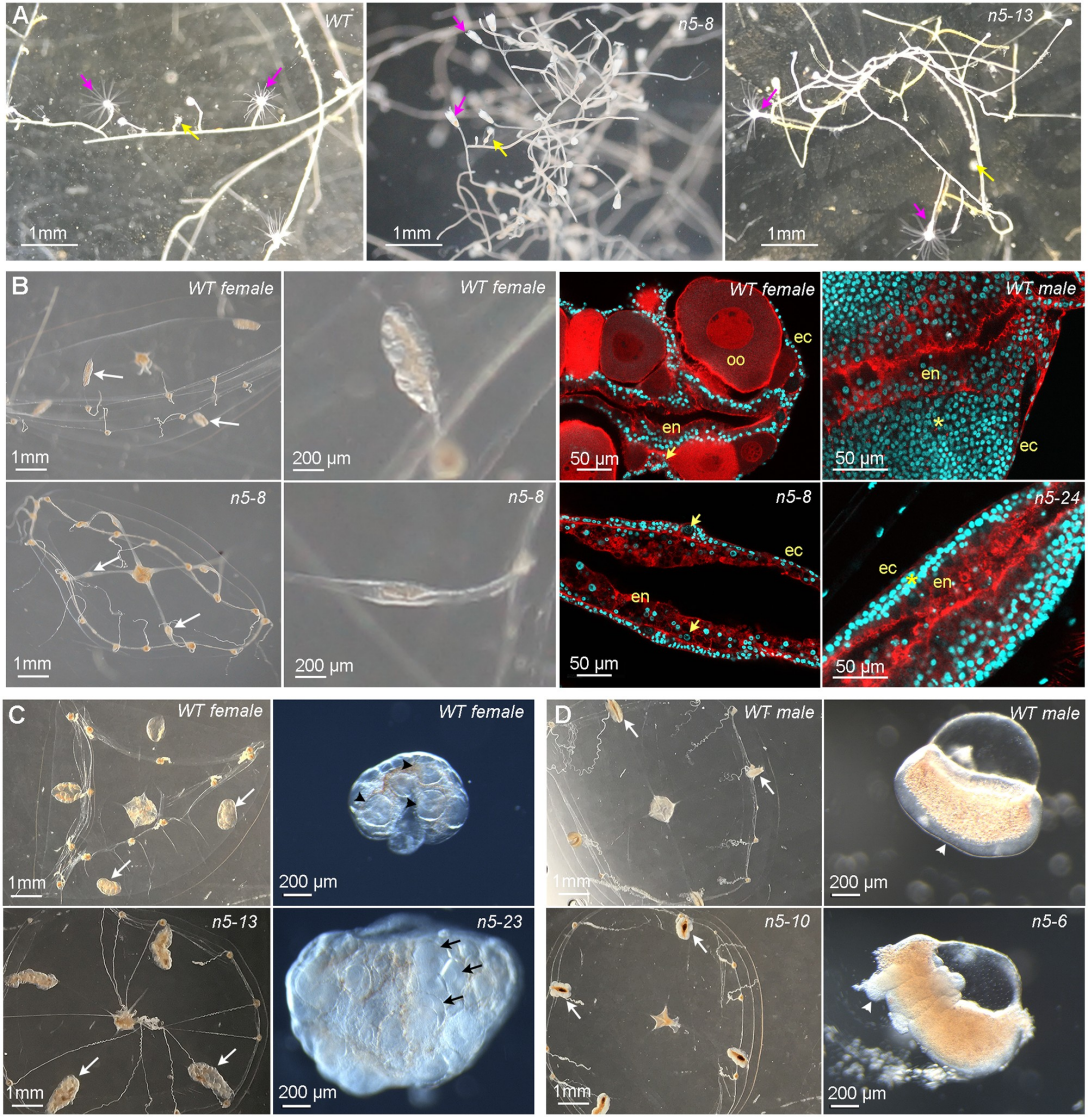
Phenotypes of *Clytia MIHR* mutants. Light and confocal microscope images of *MIHR* mutant F0 polyp colonies and jellyfish. (A) Morphology of a wild-type (WT) colony, Z11, and two *MIHR* mutant colonies (n5-8 and n5-13), as indicated. All *MIHR* mutant colonies contained gastrozooid and gonozooid polyps (pink and yellow arrows, respectively); however, the connecting stolons in some mutant colonies (see Table 1) were convoluted and frequently detached from the glass substrate, while stolons of WT colonies and the other mutant colonies were straight and adhered tightly. (B) Phenotypes of fully grown WT (top row) and mutant n5-8 or n5-24 (bottom row) jellyfish. Mutant and WT jellyfish show very similar morphology, but gonads developed poorly in these mutants, as shown by white arrows in the first column and at higher magnification in the second column. The third and fourth columns are confocal microscope images through the gonads of adult jellyfish from females and males, respectively; nuclei are stained with Hoechst 33342 (cyan) and F-actin with Phalloidin (red). In the n5-8 female gonad, small oocytes (arrows) can be detected between the endodermal (en) and ectodermal (ec) layers, but no large growing oocytes (oo) are present compared to the WT female gonad. In the n5-24 male gonad, the spermatogenic zone (asterisk) between endoderm and ectoderm is much thinner than in the WT male gonad. (C) Comparison of mutant n5-23 and n5-13 (bottom row) female medusae gonads (white arrows) swollen by an accumulation of large oocytes to WT (Z11) female medusae (top row). Right panels show gonads dissected from 3-week-old n5-23 medusae 10 hours after a light cue that induced spawning in the WT but not the mutant gonad. Black arrowheads indicate large growing oocytes in the WT gonad and black arrows indicate fully grown oocytes in the mutant gonad. (D) Adult mutant n5-10 and n5-6 male medusa (bottom row) compared to WT (Z13) male medusae (top row) have deformed gonads (white arrows). Right panels show isolated gonads, illustrating the thickened and irregular spermatogenic layer (arrowheads) in the mutant. Scale bars as indicated. *MIHR*, MIH receptor.

To determine the function of MIHR in vivo, we generated *Clytia MIHR* knockout (KO) polyp colonies using CRISPR/Cas9 mutagenesis (see Methods). This gene-editing technique is very effective in *Clytia*, allowing extensive bi-allelic mutation of target genes already in F0 polyp colonies [16]. Three guide RNAs designed to target the third transmembrane domain of the MIHR protein were tested by genotyping at the planula stage and the most effective one used to generate polyp colonies. After genotyping around the target site, we selected and propagated for phenotypic analysis six colonies showing a very low proportion or no detectable wild-type sequence and carrying mainly frameshift mutations (Table 1). All six mutant polyp colonies expanded slowly compared to wild-type colonies. They displayed variable morphologies, with two of them rambling pronouncedly away from the glass substrate (Fig 2A; Table 1), but all produced active gonozooids that budded baby medusae. Growth of these mutant baby medusae was slower for the six mutants than for wild types, but they were able to reach the full adult size of about 1 cm in diameter. The adult jellyfish all swam less vigorously than wild types. The slow growth of mutant polyps and medusae was not due to any obvious feeding problems: both forms could capture *Artemia* nauplii without difficulty.

**Table 1:** Characteristics of *MIHR* mutant colonies.

Colony name	Predominant mutations (minor mutations)^#^	Medusa sex	Polyp colony*	Medusa gonads
n5-23	−4 nt, +74 nt (−8 nt)	Female	normal	Enlarged and deformed
n5-8	−7 nt (+11 nt)	Female	rambling	Poorly developed
n5-13	−4 nt, −3 nt	Female	rambling	Enlarged and deformed
n5-24	−34 nt, +59 nt	Male	normal	Poorly developed
n5-10	−4 nt (−6 nt, +2 nt)	Male	normal	Enlarged and deformed
n5-6	−4 nt, −3 nt (−2 nt)	Male	normal	Enlarged and deformed

^**#**^Large deletions (−) and insertions (+) were detected by gel electrophoresis of PCR fragments amplified from genomic DNA (see Methods) and Sanger sequenced. They were recognized as major PCR bands of non-wild-type size. For PCR fragments migrating close to the predicted wild-type size, local nucleotide insertions and deletions were detected by TIDE analysis (see Methods). Genotypes were classed as “predominant” if they correspond to major PCR bands from gel analysis or were estimated by TIDE analysis as more than 20% of the genotype, and as “minor” if estimated at between 5% and 20% in TIDE analysis. Any additional local mutations estimated at under 5% by TIDE analysis are not included.

*See Fig 2A.

Abbreviations: *MIHR*, MIH receptor; TIDE, Tracking of Indels by Decomposition

Of the six *MIHR* mutant colonies, three produced male jellyfish and three females. In addition to the shared defects in growth and swimming behavior, they showed one of two distinct phenotypes affecting gonad development. For one female and one male *MIHR* mutant (n5-8 and n5-24, respectively), the gonads of the adult jellyfish developed poorly, failing to accumulate gametes as occurs during wild-type adult growth (Fig 2B). Examination of the gonads by confocal microscopy revealed the presence of small oocytes for n5-8 and a thin spermatogenic zone for n5-24. This suggested that mutation of the *MIHR* gene may directly or indirectly compromise gametogenesis in male and female jellyfish. In marked contrast, jellyfish from the four other *MIHR* mutant polyp colonies underwent gametogenesis to produce large numbers of fully grown oocytes (n5-13, n5-23; Fig 2C) or spermatozoids (n5-6, n5-10; Fig 2D). In the female n5-13 and n5-23 medusae, gonads were grossly inflated by accumulation of fully grown immature oocytes. As detailed below, we could demonstrate that this was due to failure in light-induced oocyte maturation and subsequent release of the unfertilized egg, and reflected inability of the oocytes to respond to MIH, rather than a nonspecific oocyte maturation defect. In males, the gonads were markedly deformed and irregular. Following dark to light transitions that induced spawning in wild-type males, some local sperm release was observed from rupture sites of the gonad epithelium, but the sperm remained concentrated at the gonad surface and failed to disperse.

The origin of the two opposite gonad defects observed in jellyfish from different mutant *MIHR* colonies, i.e., poorly developed versus swollen with gametes, is not clear. There was no obvious relationship between gonad phenotype and genotype at the CRISPR target site (Table 1). All mutations detectable at above 5% in genomic DNA extracted from the colonies were frameshift, with the exception of minor 3-nt and 6-nt deletions in colonies n5-13 and n5-10, respectively. The possibility remains that in some of the colonies, small populations of cells with non-frameshift mutations, or residual wild-type cells, could directly or indirectly facilitate gonad development. We also cannot rule out that the poor gonad development in *MIHR* mutants n5-8 and n5-24 results from off-target mutations induced by Cas9, even though we prescreened the CRISPR target sites against the *Clytia* genome (see Methods). On the other hand, off-target mutations are unlikely to explain the oocyte maturation failure, which could be selectively reversed by treatment with cAMP analogues (see below). We favor the explanation that the poor overall growth of the jellyfish observed in all *MIHR* mutants affected gamete growth within the gonads more severely in some strains than others, perhaps due to variation in their genetic backgrounds.

This initial phenotype analysis of jellyfish from six independent *MIHR* mutant F0 polyp colonies suggests both global and gonad-specific roles for MIHR. Firstly, it may have nonessential functions in regulating medusa swimming and medusa growth that may impair gametogenesis in some cases, although the specificity of these phenotypes is not fully confirmed. Secondly, it has a specific role in regulating gamete maturation and release. For females, the overaccumulation of immature, fully grown oocytes in medusae from two independent *MIHR* mutants strongly supported the identity of this GPCR as the MIH receptor. Its role in oocytes is characterized further below.

### *Clytia* MIHR is expressed in oocytes and also in tentacle cells

**Fig 3:**
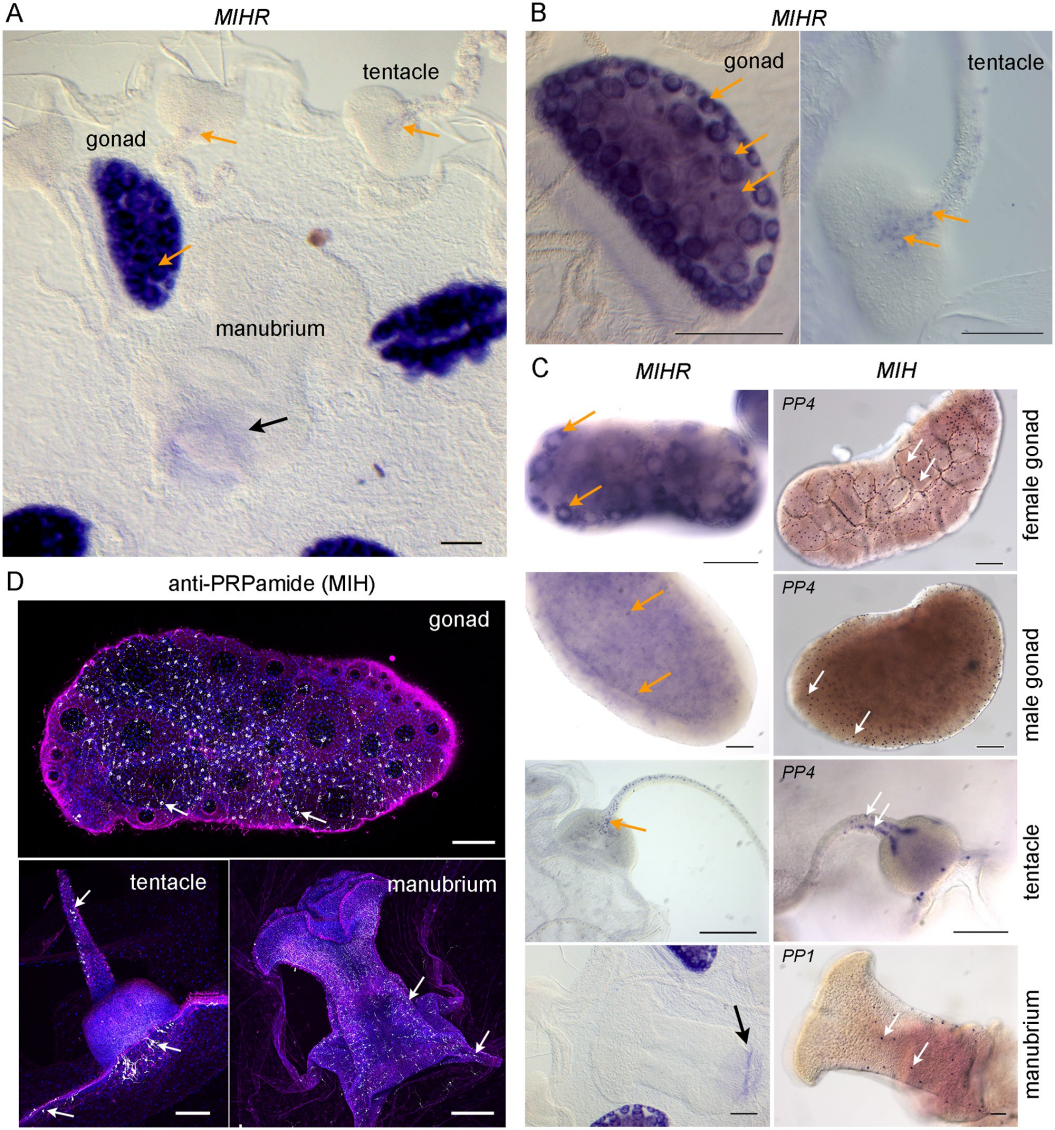
Sites of *MIHR* expression in the *Clytia* medusa. (A) In situ hybridization detection of *MIHR* mRNA in a young adult *Clytia* female medusa. Strong purple *MIHR* signal (orange arrows) was detected in oocytes within the gonads, as well as in scattered cells in tentacles. (B) Higher magnification images of MIHR mRNA detected in young adult medusae; with the gonad, each oocyte (e.g., at orange arrows) has an unstained nucleus. The intensity of labelling in individual oocytes decreases as they grow due to dilution of cytosol with yolk. In the tentacle, a row of individual MIHR-positive putative neural cells can be clearly distinguished leading from the center of the bulb on its oral face. (C) Comparison of the distribution of the MIH receptor and ligand expressing cells in different medusa structures as labelled, detected by in situ hybridization using probes to *MIHR* (top row) and to the MIH peptide precursors *PP1* and/or *PP4* as indicated (bottom row). Orange arrows point to oocytes, developing spermatozoa and tentacle MIHR cells, and white arrows indicate MIH cells. The focal plane in the male gonad image is through the center to illustrate the position of the MIH cells in the ectodermal layer. Weak staining at the base of the manubrium (black arrow) in A and B is frequently observed with probes for many genes and is probably due to a specific trapping of the color reagent. Scale bars: 100 μm. (D) Confocal images of the three main sites of MIH-expressing cells (white arrows) in medusae, visualized using anti-PRPamide antibody (MIH: white), anti-tyrosinated tubulin (magenta), and Hoechst staining of nuclei (blue). Summed z-stacks are shown in all cases except for the gonad tubulin and DNA staining, where a single plane was selected through the center of the gonad. All scale bars: 100 μm. MIH, maturation-inducing hormone; *MIHR*, MIH receptor.

The observed defects in *MIHR* mutant jellyfish suggested that this receptor may have roles both in the gonads and other sites. In situ hybridization detection of *MIHR* mRNA in adult jellyfish (medusae) and in isolated gonads (Fig 3) supported this idea. Strong expression was detected in developing oocytes and in male gametes, consistent with the ability of MIH to induce male spawning [15]. Expression was also detected in clusters of small somatic cells located within each tentacle bulb. Individual cells from these clusters extended in a line along each tentacle (Fig 3A, 3B and 3C). MIHR in tentacle cells could potentially respond to MIH neuropeptides produced by neural/neuroendocrine cells of the endodermal gastrovascular system, detectable using an antibody recognizing the PRPamide peptides produced from both Che-PP1 and Che-PP4 (anti-PRPa [15]; Fig 3D). These two precursors are expressed respectively in association with the manubrium and tentacle, as well as being co-expressed in scattered cells of the gonad [15]. Comparison of in situ hybridization patterns for *MIHR* and *MIH* showed that the ligand and receptor-expressing cells in the tentacles have distinct distributions: the single file of MIHR cells lies on the rounded oral side [31], whereas the MIH cells form two flanking lines (Fig 3B and 3C). No *MIHR*-expressing cells were detected in the manubrium, only nonspecific staining of the manubrium floor. Regulation of tentacle contractions by MIHR-expressing cells in the tentacles in response to MIH produced in the gastrovascular system, integrated in a wider neuronal system, for instance including RFamide expressing cells [32], could account for the sluggish movement and poor growth of *MIHR* mutant medusae. Specific assays should be devised in the future to examine this hypothesis in detail.

### *Clytia* MIHR is the essential oocyte receptor for oocyte maturation

**Fig 4:**
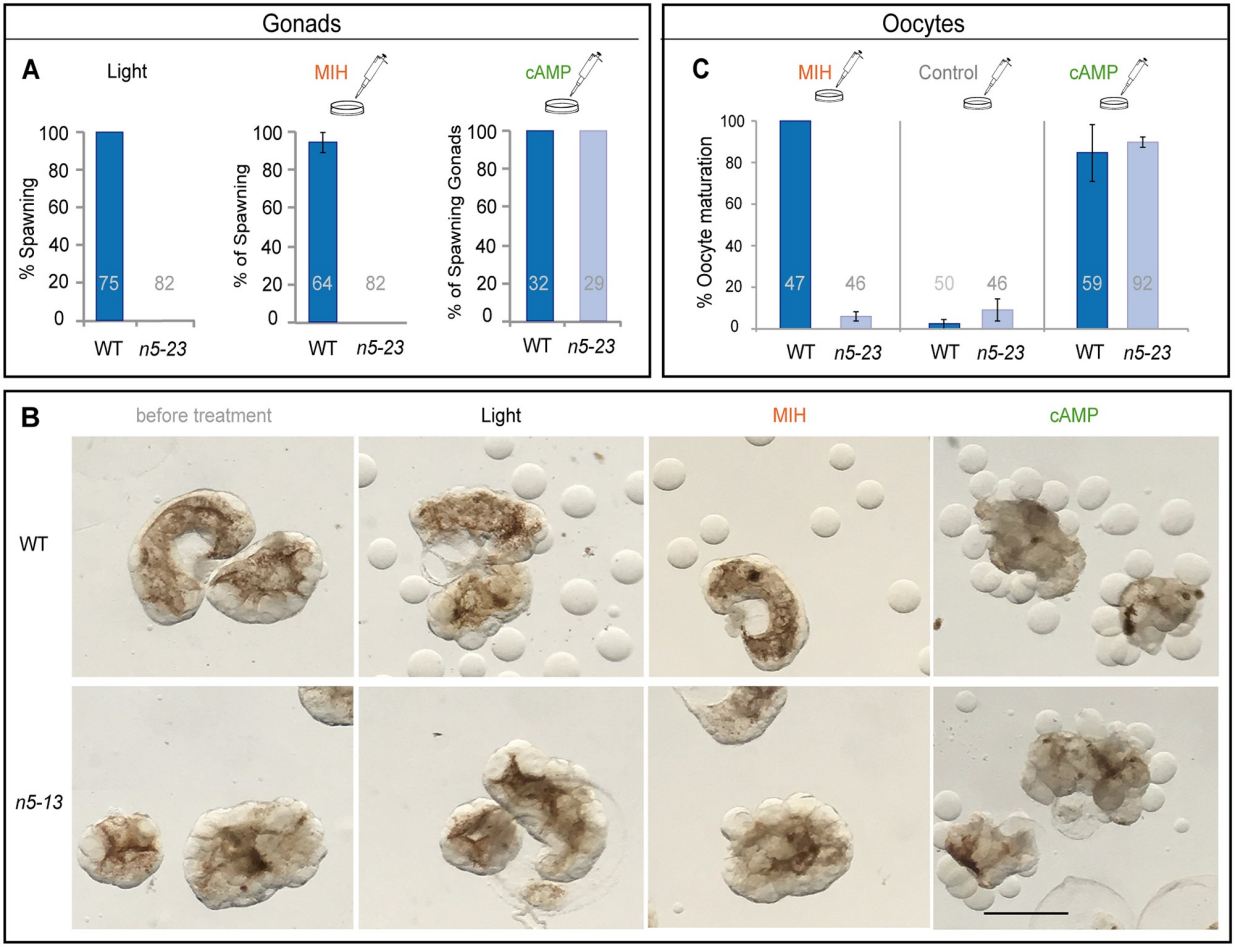
Oocyte maturation failure in *MIHR* mutant medusae. Oocyte maturation assays performed using isolated gonads (A, B) or isolated oocytes (C) from *MIHR* mutant jellyfish compared to WT. In A and C, bar heights represent mean percentages of 3 independent experiments and error bars show standard deviations. Total gonad or oocyte numbers for each treatment are indicated in gray. (A) Spawning response of isolated gonads from WT and n5-23 female medusae. Three treatments were compared as indicated above each panel: Light: light stimulation after incubation in the dark; MIH: treatment of light-maintained gonads with 100 nM WPRPamide; cAMP: treatment of light-maintained gonads with 4 mM Br-cAMP. No oocyte maturation or spawning was observed in *MIHR* KO gonads upon light stimulation or MIH treatment, while Br-cAMP treatment provoked oocyte maturation and spawning. The Fisher exact test showed significant differences (F = 0) between wild-type and mutant responses to light and MIH, but not for the cAMP treatment (F = 1). Full datasets from three independent experiments are available in S3 Data. (B) Light microscope images illustrating gonads from an equivalent experiment performed with n5-13 female *MIHR* mutant medusae 120 minutes after the indicated treatments. Scale bar: 500 μm. (C) Response of fully grown oocytes isolated from WT and n5-23 *MIHR* mutant gonads to MIH and Br-cAMP treatments as in A. Both treatments triggered maturation of WT oocytes, visible after 20 to 30 minutes as germinal vesicle breakdown (GVBD), but only Br-cAMP induced maturation of *MIHR* KO oocytes. Control experiments using the Br-cAMP solvent (distilled water) showed a low level of spontaneous maturation in both cases. The Fisher exact test did not show significant differences between WT and mutant oocytes in the control (F = 0.101) or cAMP-treated (F = 0.216) groups, but did so in the MIH assays (F = 0). Full datasets from 3 independent experiments are available in S3 Data. Br-cAMP, bromoadenosine 3′,5′-cyclic monophosphate; cAMP, cyclic adenosine monophosphate; KO, knockout; MIH, maturation-inducing hormone; *MIHR*, MIH receptor; WT, wild-type.

We used jellyfish from the two *MIHR* female mutant colonies that showed the swollen-gonad phenotype (n5-13 and n5-23) to characterize the role of this GPCR in oocyte maturation. We performed a series of oocyte maturation and spawning assays, comparing the responses of wild-type and *MIHR* mutant isolated gonads and oocytes to different stimuli (Fig 4). Isolated wild-type gonads underwent oocyte maturation and spawning in response to light stimulation or treatment with the synthetic MIH peptide WPRPamide (100 nM) as previously shown [15], but *MIHR* mutant gonads did not respond (Fig 4A and 4B). In contrast, treatment with 4 mM bromoadenosine 3′,5′-cyclic monophosphate (Br-cAMP), a cell-permeable analogue of cAMP that induces hydrozoan oocyte maturation by mimicking the early cytoplasmic cAMP rise [3,21], rescued the phenotype of *MIHR* mutant gonads, efficiently triggering oocyte maturation and spawning. Br-cAMP treatments but not MIH peptides also promoted maturation of isolated fully grown *MIHR* mutant oocytes (Fig 4C). Br-cAMP–matured *MIHR* mutant oocytes could be fertilized to develop into planula larvae, although they had lower development and metamorphosis rates than wild-type oocytes. This demonstration of a cAMP-reversible maturation initiation failure in mutant female jellyfish confirmed that the *Clytia* MIHR has an essential in vivo function as the oocyte receptor for MIH.

### MIH-induced oocyte maturation blocked by an inhibitory Gα_s_ antibody

**Fig 5:**
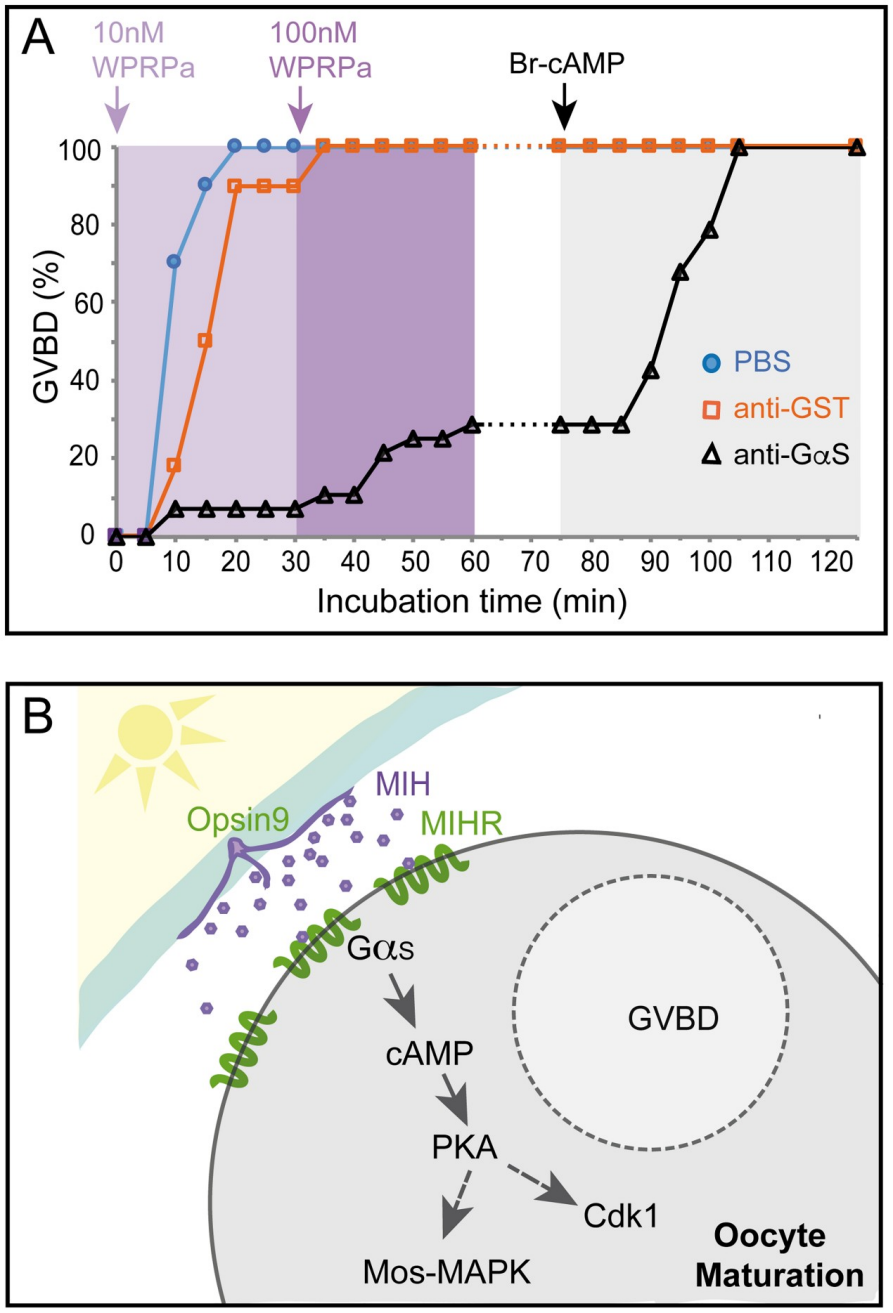
Involvement of Gα_s_ in *Clytia* oocyte maturation. (A) Results of an antibody inhibition experiment. Maturation response (scored as percent GVBD over time) of isolated oocytes injected with antibodies or buffer, then challenged first with a low dose of MIH (10 nM WPRPamide), then with a higher dose (100 nM WPRPamide), and finally with Br-cAMP to verify maturation competence, as indicated by the colored arrows and background shading. Oocytes injected with PBS (blue circles) or a control anti-GST antibody (orange squares) responded efficiently to MIH, whereas very few oocytes injected with anti-Gα_s_ (black triangles) underwent GVBD after treatment with the low dose of MIH and only 29% following the high dose. The number of oocytes per group in this experiment was 30, 28, and 30, respectively. All of them had undergone GVBD by the end of the experiment. Times from the start of the first incubation are shown on the x-axis. Full datasets from five independent experiments are available in S4 Data. (B) Scheme illustrating the proposed cascade initiating *Clytia* oocyte maturation initiation. Following light stimulation after a dark period, Opsin9 mediates release of MIH neuropeptides from specialized cells (purple) of the gonad ectoderm (cyan). Activation of the MIHR (green) at the oocyte surface releases Gα_S_ to promote an increase in cytoplasmic cAMP, activating PKA. Unknown PKA substrates likely trigger in parallel Cdk1 activation and thus GVBD, and Mos1 synthesis to initiate the MAPK cascade.; Br-cAMP, bromoadenosine 3′,5′-cyclic monophosphate; GST, Glutathione-S-Transferase; GVBD, germinal vesicle breakdown; MIH, maturation-inducing hormone; MIHR, MIH receptor; PKA, protein kinase A.

GPCR activation can lead to a cytoplasmic cAMP concentration rise in the responding cell as a result of adenylate cyclase stimulation via S type Gα subunits of heterotrimeric G proteins. We tested the role of Gα_s_ in *Clytia* oocyte maturation by injecting isolated oocytes with a specific inhibitory antibody previously shown to cause meiotic maturation of mouse, *Xenopus*, and zebrafish oocytes [12,33,34]. All oocytes were tested for maturation competence by incubation at the end of the experiment in Br-cAMP. Any that failed to mature when treated with Br-cAMP were discounted in the analyses. Oocytes injected with anti-Gα_s_ responded less efficiently than oocytes injected with PBS or a control anti–Glutathione-S-Transferase (GST) antibody when treated with synthetic MIH at low and then high doses (10 nM then 100 nM WPRPamide; one of three equivalent experiments documented in Fig 5A). These results strongly suggest that MIHR acts mainly through Gα_s_.

Based on these findings, we can now propose a model for maturation initiation in *Clytia* oocytes through MIH, MIHR, Gα_s_, and cAMP (Fig 5B). A light cue triggers MIH release from neuroendocrine-type cells in the gonad ectoderm via an essential opsin protein [16]. The MIH peptides act on MIHR at the oocyte surface to promote Gα_s_ activation, stimulating adenylate cyclase and thus promoting an increase in cytoplasmic cAMP concentration, essential for the transition into meiotic M phase.

### *Clytia* MIHR is related to a bilaterian superfamily of neuropeptide hormone receptors

**Fig 6:**
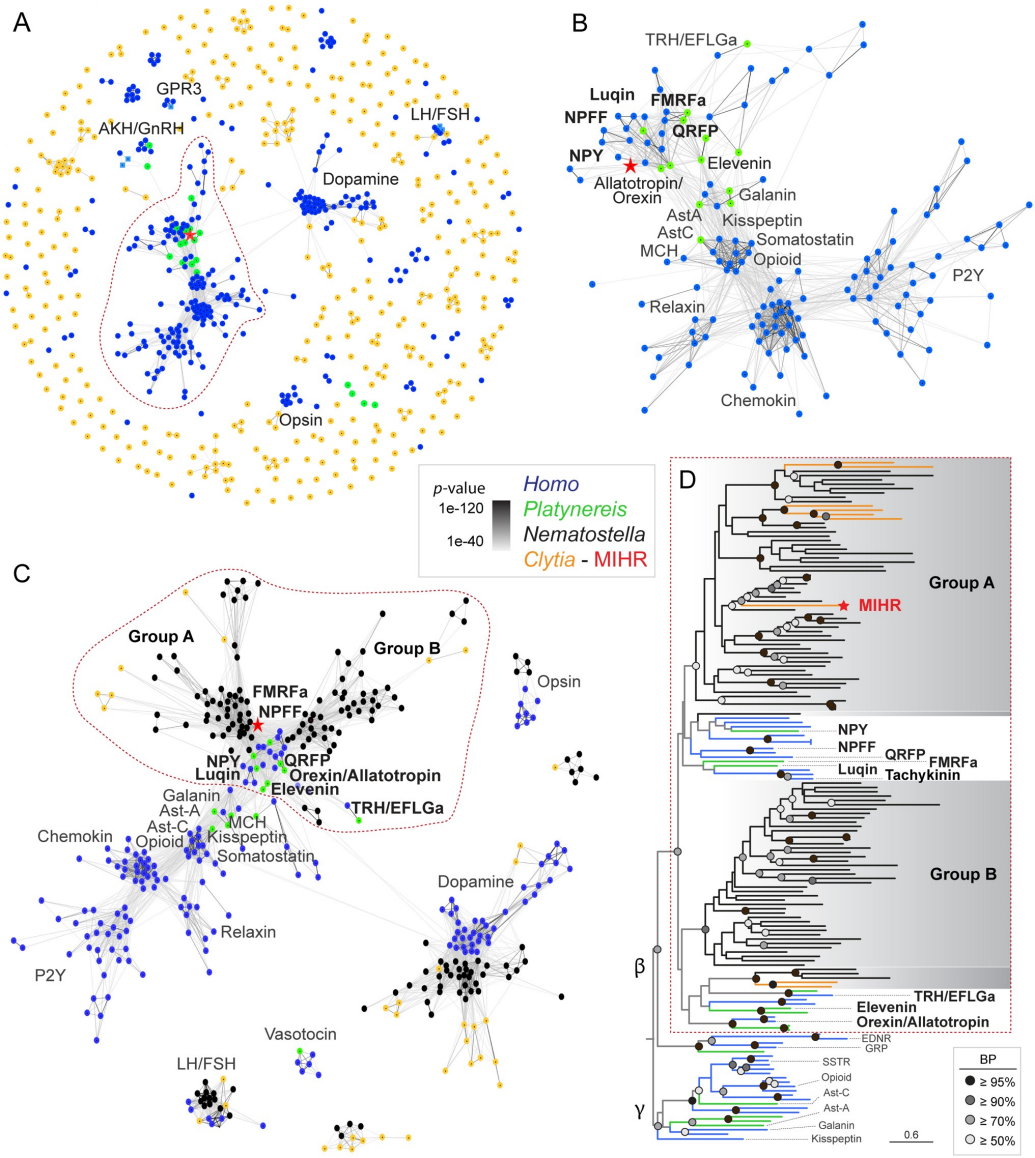
Relationship of *Clytia* MIHR to bilaterian neuropeptide hormone GPCRs. (A) Sequence-similarity-based clustering using Clans2 of all identified class-A GPCRs from *Clytia*, human (olfactory receptors excluded), and *Platynereis* deorphanized GPCRs [28] BLASTP *p*-value < 1e-40. (B) Cluster map of the largest cluster (circled in red in A), keeping only sequences that show at least 2 connections with the central cluster. BLASTP *p*-value < 1e-40. (C) More stringent cluster map (*p*-value < 1e-50) of the same sequences as in (A) plus all *Nematostella* GPCR-A sequences. Only clusters containing at least 5 sequences from at least 2 species were kept. All connections with *p*-value <1e-40 are shown. (D) Maximum likelihood analysis of the sequences contained inside the dashed area shown in (C) using RaxML (PROTGAMMAGTR) with 500 Bootstrap replicates (BR). Rhodopsin *beta* GPCRs are rooted against rhodopsin *gamma* GPCRs [37]. Tree file provided in S2 Fig. Color code: *Homo sapiens*: blue, *Platynereis dumerilli*: green, *Nematostella vectensis*: black, *Clytia*: orange. Red star: *Clytia* MIHR. FSH, follicle-stimulating hormone; GnRH, gonadotropin-releasing hormone; GPCR, G protein–coupled receptor; GPR3, G protein–coupled receptor 3; LH, luteinizing hormone; MIHR, MIH receptor; NPY, neuropeptide Y; P2Y, purinoceptor; QRFP, pyroglutamylated RFamide peptide.

Much progress has been made in understanding the relationships between GPCR-neuropeptide families between protostomes and deuterostomes [20,25,28,35,36], and more recently with those in acoels and cnidarians [37]. We performed sequence-similarity-based clustering to explore the relationship of the *Clytia* MIHR sequence with known GPCR families in Bilateria using established datasets from human and the annelid *Platynereis* [25,28] (Fig 6A). The *Clytia* MIHR fell within the majority group of cnidarian GPCRs that cluster with a superfamily of peptide hormone receptors, including human neuropeptide Y, neuropeptide FF, tachykinin, orexin, elevenin, and EFLGa/thyrotropin-releasing hormone, as well as luqin from *Platynereis* (Fig 6B). Adding to this analysis sequences recovered from the anthozoan cnidarian *Nematostella* highlighted the extensive independent expansion of the GPCR-A family since cnidarian and bilaterian neuropeptide systems split [37]. This analysis demonstrated that the MIHR group does not contain any of the GPCRs directly involved in controlling oocyte maturation in vertebrates, including the constitutively active GPR3, gonadotropin-releasing hormone (GnRH) receptor, or luteinizing hormone (LH)/follicle-stimulating hormone (FSH) receptor (see Introduction). A GPCR from *Nematostella* annotated as a GnRH receptor [38] associates with a distinct "superfamily" including the bilaterian GnRH, vasotocin, CCAP, corazonin, and achatin receptor families.

Maximum likelihood phylogenetic analyses of the large GPCR cluster containing MIHR confirmed phylogenetic support for a GPCR "superfamily" associating two distinct cnidarian groups (groups A and B in Fig 6C and 6D) with a set of bilaterian neuropeptide hormone receptors [37]. Cnidarian GPCR group A contained *Clytia* MIHR, six other *Clytia* GPCRs, and a large number of *Nematostella* receptors. It showed weakly supported association with a set of bilaterian peptide hormone receptor families, including those for neuropeptide Y/neuropeptide F, gonadotropin inhibitory hormone (GnIH)/neuropeptide FF, RYa/Luqin, tachykinin, and pyroglutamylated RFamide peptide (QRFP) [25,28,37]. Group B included two *Clytia* GPCRs and associated with further bilaterian neurohormonal receptor families, including elevenin, EFLGa/TRH, and orexin/allatotropin receptors (Fig 6C and 6D). The low support values for many deep branch relationships within this superfamily make it difficult to determine definitive evolutionary relationships between the neuropeptide hormone receptors and cnidarian groups A and B GPCRs. We can nevertheless conclude that *Clytia* MIHR, along with eight other *Clytia* GPCRs and a very large group of *Nematostella* GPCRs, have a common ancestor with a subset of bilaterian peptide hormone receptor families that includes many involved in regulating sexual reproduction and feeding.

## Discussion

GPCR deorphanization followed by targeted gene mutation allowed us to identify *Clytia* MIHR as the neuropeptide receptor responsible for initiating oocyte meiotic maturation. *MIHR* female mutant medusae accumulated fully grown oocytes that failed to mature unless treated with cAMP analogues to bypass the receptor. We further provided evidence that a Gα_s_ subunit links MIHR activation to the increase in cytoplasmic cAMP concentration in the oocyte that initiates maturation. MIHR belongs to a superfamily of neuroendocrine GPCRs involved in regulating reproduction but also nutrition, as well as diverse other physiological processes. *Clytia* MIH and MIHR are expressed in the gastrovascular system, tentacles, and possibly elsewhere and likely have other roles than in gamete maturation. Our findings thus open doors to an improved understanding of the vital animal process of oocyte maturation and help to shed light on the evolution of the neurohormonal regulation of sexual reproduction.

### An oocyte receptor for maturation initiation

Our work shows that *Clytia* MIHR is entirely responsible for the oocyte response to MIH, allowing us to fill in the main molecular actors leading to oocyte meiotic maturation (Fig 5B): MIH is released from specialized gonad ectoderm cells in response to a dark to light transition at dawn via the essential photoresponsive GPCR Opsin9 [16]. Binding of MIH to MIHR at the oocyte surface is likely swiftly followed by a rise in cytoplasmic cAMP levels in the oocyte to activate cAMP-dependent protein kinase (PKA), as has been shown in other hydrozoan species [22]. Our antibody inhibition experiments strongly implicate Gα_s_-stimulated adenylate cyclase activity in driving this cAMP rise. PKA leads to activation of the Cdk1-CyclinB and Mos-MAP kinase systems, which have highly conserved roles in oocyte maturation [39,40]. To complete the full chain of events between MIH secretion and meiotic maturation will require the identification of PKA substrates from *Clytia* oocytes that interact with regulators of Mos translation and/or Cdk1-CyclinB autoactivation.

cAMP rises initiate oocyte maturation not only in hydrozoans but also in a diverse range of protostome and deuterostome species [6], so it is tempting to speculate that the straightforward pathway linking MIH secretion to meiotic maturation in *Clytia* may have retained its main features from a distant cnidarian-bilaterian ancestor. A useful step towards testing this idea would be to search for MIHR superfamily GPCRs in oocytes of nemertean, ascidian, bivalves, or ophiuroid species that depend on cAMP signalling for maturation. If confirmed as the ancestral state, the *Clytia* MIH-MIHR system could provide a paradigm for the molecular dissection of a common, cAMP-stimulated mechanism for oocyte meiotic resumption.

### Evolution of reproductive regulation

**Fig 7:**
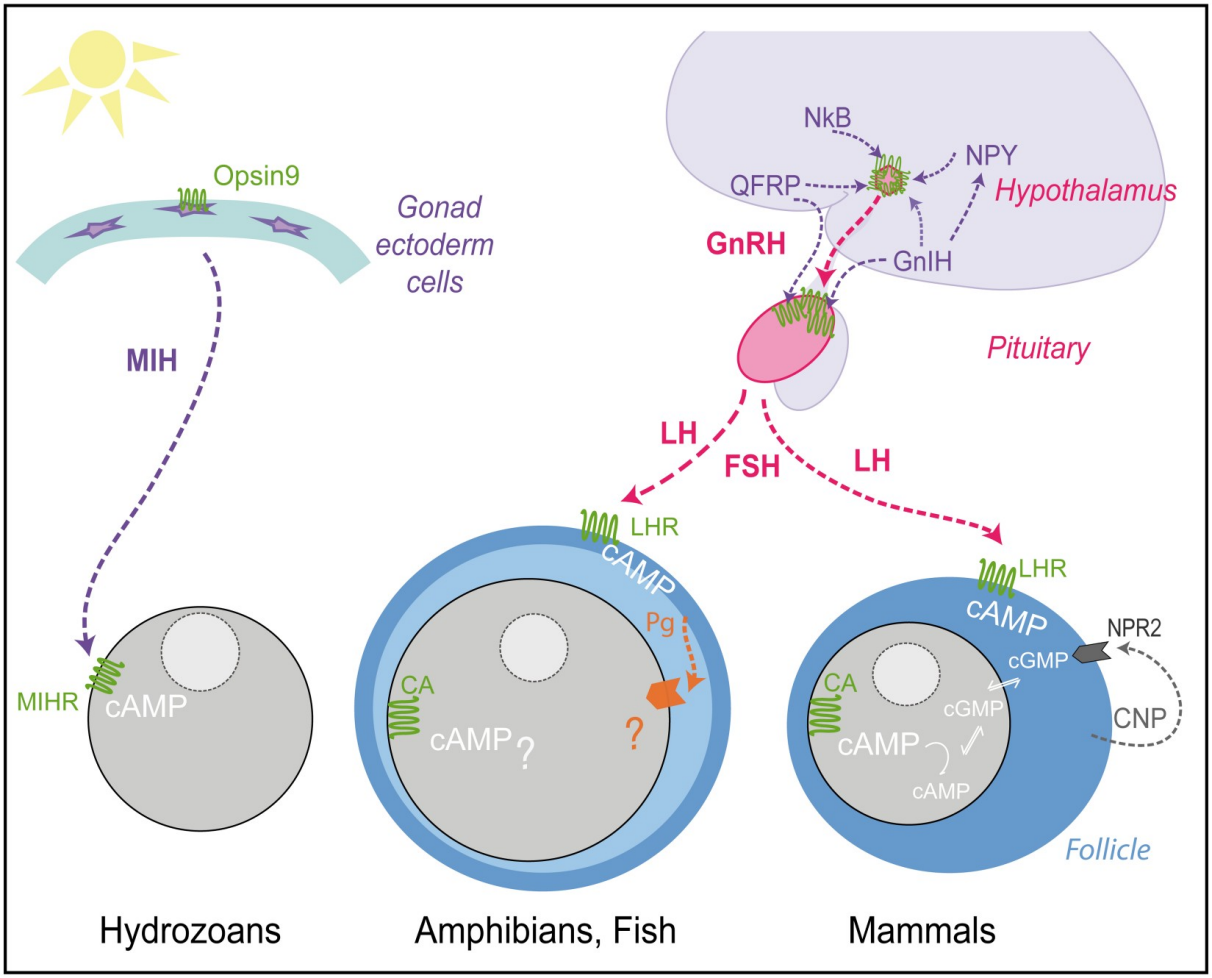
Schematic comparison of GPCR regulation of *Clytia* and vertebrate oocyte maturation. Simplified view of the tissues, hormones, and receptors involved in regulating oocyte maturation in *Clytia* and in fish/amphibians and mammals. For simplicity, we have not included protostome or echinoderm models. The principle peptide hormones of the reproductive hypothalamus-pituitary-gonadal axis (GnRH and LH/FSH) are in pink, and those for which the receptors group phylogenetically with *Clytia* MIHR in “Group A” (Fig 6) are in purple. Peptide hormones: *Clytia* MIH, Neuropeptide Y (NPY), GnIH, GnRH, LH, QRFP, NkB, and C-type natriuretic peptide (CNP). All their receptors, except the guanylyl cyclase natriuretic peptide receptor 2 (NPR2) activated by CNP, are GPCRs (green). Constitutively active (CA) GPCRs in vertebrate oocytes maintain cytoplasmic cAMP levels high prior to maturation. In mouse oocytes, a cAMP decrease upon hormone stimulation triggers maturation; however, in fish and frog oocytes the degree and role of this decrease is debated. Several types of oocytes receptor (orange) may respond to steroid hormones (Pg) in different species of amphibians and fish, but the relative importance of multiple downstream signalling pathways remains to be clarified [1,43,44,45,46]. See text for discussion. CNP, C-type natriuretic peptide; FSH, follicle-stimulating hormone; GnIH, gonadotropin inhibitory hormone; GnRH, gonadotropin-releasing hormone; GPCR, G protein–coupled receptor; LH, luteinizing hormone; LHR, lutenizing hormone receptor; MIH, maturation-inducing hormone; MIHR, MIH receptor; NkB, neurokinin B; QRFP, pyroglutamylated RFamide peptide.

The role of cAMP in initiating oocyte maturation is not universal. In some cases, such as in starfish, it has no significant role [41], while in many vertebrate species, high cAMP levels in the ovarian oocyte have been strongly implicated in maintaining the prophase arrest. Clues to understanding how these marked differences arose during evolution may be found by considering the GPCRs involved in regulating gamete maturation and release across species. In vertebrates, GPCRs are involved in triggering oocyte maturation at several levels along the hypothalamus-pituitary-gonadal axis (Fig 7). At the level of the ovarian oocyte, constitutively active GPCRs of the GPR3/6/12 family help maintain prophase arrest via Gα_s_-cAMP [12–14]. LH receptors in the surrounding follicle cells provide upstream signalling also via Gα_s_-cAMP. The follicle cells signal is transmitted to the oocyte in mouse via a cGMP decrease through gap junctions [42]. In fish and amphibians, follicle cells secrete steroid hormones that act on one or more types of oocyte membrane receptor. The extent and importance of the cAMP drop observed at maturation initiation vary in different systems studied, and other signaling pathways are likely to be important, at least in some species [39,40,43,44,45,46]. Upstream of the vertebrate follicle, LH production from the pituitary is under control of the master reproductive regulator GnRH receptor acting via Gα_q_ and cytoplasmic Ca^++^ release. Many other hormone-GPCR pairs influence production of GnRH by the hypothalamus, as well as the gonadotropins LH and FSH by the pituitary. Molecular phylogeny showed that *Clytia* MIHR is not closely related to receptors of the core hypothalamus-pituitary-gonadal GPCRs, but rather forms a superfamily with receptors of these “upstream” neuropeptide hormones, notably GnIH, QRFP, Neuropeptide Y, and NkB. Production of GnRH and gonadotropins along the hypothalamus-pituitary-gonadal axis are inhibited by GnIH and stimulated by QRFPs [47,48], while the tachykinin family hormone NkB acts in a group of hypothalamus neurons together with kisspeptin and dynorphin A in the generation of cyclic GnRH pulses [49]. The ancestral cnidarian-bilaterian GPCR for MIHR and the receptors to all these vertebrate hormones may thus already have had a role in regulating gamete production and/or release. In this case, vertebrate GPCRs in the hypothalamus and the pituitary, rather than those in gonad follicle cells or gametes, would have retained the trace of this ancestor. Likewise, protostome members of the MIHR GPCR group regulate sexual reproduction indirectly from sites far from the gonad. The planarian neuropeptide Y NPY-8 regulates gametogenesis through its GPCR expressed in central nervous system neuroendocrine hormone cells [50], while nematode luqin peptides produced by pharyngeal neurons regulate egg laying via serotonergic RIH interneurons [51].

### An ancient regulatory system linking reproduction and nutrition?

It is striking that many bilaterian neuropeptide GPCRs closely related to *Clytia* MIHR are known for roles in regulating feeding and nutritional balance. In mammals, these include GPR83/PEN [52] as well as QRFP receptors and neuropeptide Y receptors, which provide important links between metabolic state and reproductive regulation [53–55], in conjunction with GnIH signalling [56]. Related hormone receptor families from protostomes are also known for regulating feeding, including those for *Drosophila* leucokinin [57] and luqins in arthropods (RYamides) and nematodes [51,58]. It is thus tempting to propose that the ancestral GPCR of the *Clytia* MIHR and these bilaterian hormone receptor families was involved in integrating sexual reproduction with nutritional status. It should be noted, however, that bilaterian neuropeptide GPCRs in this superfamily also function in many other physiological processes. Conversely, many neuropeptide hormone systems involved in integrating sexual reproduction with feeding, notably including kisspeptin, involve GPCRs only distantly related to MIHR [35,37,59]. To unravel further the evolutionary history of hormonal regulation of gamete production in relation to nutrition, it will be of great interest to address the non-oocyte functions of MIHR in *Clytia*, and also to determine the functions of other “group A” GPCRs from *Clytia* and other cnidarian species.

## Methods

### Animals

Sexually mature jellyfish generated from laboratory maintained *C*. *hemisphaerica* polyp colonies (“Z strains”) [26] were fed regularly with *Artemia* nauplii and cultured under light–dark cycles to allow daily spawning. Red Sea Salt brand artificial seawater (ASW) was used for all culture and experiments.

### Selection of candidate *Clytia* MIH receptors

From a comprehensive *Clytia* reference transcriptome (86,606 contigs) derived from mixed larva, polyp, and jellyfish samples, we generated a list of predicted protein sequences from complete and incomplete ORFs using a homemade script [60]. This dataset was screened using TMHMM 2.0c to produce a list of *Clytia* complete protein sequences coding for 7TM proteins, including putative incomplete sequences containing 2 to 7 transmembrane domains. Scanning with Interproscan 5.22 was used to generate a list of 761 potential *Clytia* GPCRs with Pfam tags related to 7TMD receptors that were retained and sorted by class. CD-HIT [61] was run with 95% identity to eliminate sequence duplicates, obtaining a final dataset of 536 *Clytia* GPCRs (sequences in S1 Text). Illlumina HiSeq 50-nt reads from mRNA isolated from manually dissected gonad ectoderm, endoderm, growing oocytes, and fully grown oocytes [16] and from other *Clytia* life cycle stages, including polyp and planula larvae [26], were mapped against all candidate GPCR sequences using Bowtie2 [62]. The counts for each contig were normalized per total reads of each sample and contig length to allow expression comparisons between genes and samples.

Presumptive GPCR sequences were separated in groups using a hierarchical pipeline based on the correlation of their expressions (gplots package in R). Z-scores were obtained after standardization of the counts in the different sequenced samples for each GPCR candidate (S5 Data) and plotted in a heat map using R (S1 Fig). For clustering, a previous collection of GPCRs [25] was complemented with several bilaterian class C GPCRs retrieved from UniProtKB and all putative *Clytia* GPCRs. Clustering analysis was performed using CLANS2 [63] with a BLOSUM62 matrix and a *p*-value cutoff of 1e-30. Based on this information, Pfam signatures, reciprocal BLASTs, and high expression level in oocytes, a subset of 16 top candidates was manually selected (sequences and accession numbers in S2 Text).

### Candidate receptor cloning

The selected *Clytia* GPCRs were cloned from gonad-extracted cDNA into pcDNA3.1(+) (Thermo Fisher Scientific, Waltham, MA) using the Gibson Assembly Cloning Kit (New England Biolabs) [64]. pcDNA3.1(+) vector was linearized with BamHI and NotI restriction enzymes. Primers were designed using the Gibson Cloning option in Geneious v8. Forward primers consisted of the overhang left after BamHI vector linearization followed by the Kozak consensus sequence (CGCCACC), a start codon (ATG), and a sequence corresponding to the target sequence. Reverse primers consisted of the overhang left after NotI vector linearization followed by a STOP codon, and a reverse complementary sequence to the target sequence. The primers for all cloned GPCRs are listed in S1 Table. Polymerase chain reaction was performed using Phusion polymerase (New England Biolabs). Cloned GPCRs were sequenced using primers for T7: TAATACGACTCACTATAGGG and BGHrev: TAGAAGGCACAGTCGAGG.

### Receptor deorphanization

Cell culture GPCR ligand response assays were performed as described [28]. Briefly, CHO-K1 cells were cultured in Ham’s F12 Nut Mix medium (Thermo Fisher Scientific) with 10% foetal bovine serum. Cells were seeded in 96-well plates at approximately 10,000 cells/well and transfected the following day with pcDNA3.1(+) plasmids encoding each of the 16 candidate *Clytia* GPCRs (S2 Text), the promiscuous Gα-16 protein [65], and a reporter construct GFP-apoaequorin [66] (60 ng each) using the transfection reagent TurboFect (Thermo Fisher Scientific). After 2 days of expression, the medium was removed and replaced with Hank’s balanced salt solution (HBSS) supplemented with 1.8 mM Ca2+, 10 mM glucose, and 1 mM coelenterazine h (Promega, Madison, WI). After incubation at 37 °C for 2 hours, cells were tested by adding synthetic peptides (GenScript) in HBSS supplemented with 1.8 mM Ca2^+^ and 10 mM glucose. A list of all synthetic peptides used is provided in S2 Table. Luminescence was recorded for 45 seconds in a plate reader (BioTek Synergy Mx or Synergy H4; BioTek, Winooski, VT). Data were integrated over the 45-second measurement period and recorded as technical triplicates in each case. Data were normalized using the response of *Platynereis* FLamide receptor to 1 μM AKYFL-NH2 [28]. Dose–response curves were obtained using concentrations between 0.01 nM and 100 μM for each peptide. Data for dose–response curves were recorded in triplicate for each concentration and the experiment was repeated independently 3 times. Dose–response curves were fitted with a four-parameter curve using Prism 6 (GraphPad, La Jolla, CA) and were normalized to the calculated upper plateau values (100% activation).

### Generation of CRISPR-Cas9 mutant *Clytia* polyp colonies

Following our established protocol [67], *MIHR* small guide RNA (sgRNA) was assembled by hybridizing crRNA (CRISPR RNA) and tracrRNA synthesized at IDT (Integrated DNA Technologies, Coralville, IA), obtaining a final concentration of 50 μM. sgRNA was kept at −80 °C until use. The crRNA *MIHR* sequence is shown in S3 Table. We avoided off-target matches by scanning the *Clytia* genome assembly at http://crispor.tefor.net. Purified Cas9 protein in Cas9 buffer (10 mM Hepes, 150 mM KCl) provided by J-P Concordet (MNHN Paris) was diluted to 10 μM. sgRNA was added to Cas9 protein in excess (approximately 2:1) prior to injection and incubated for 10 minutes at room temperature. The final Cas9 concentration was adjusted to 4 μM and for sgRNA to 10 μM. The mixture was centrifuged at 14,000 rpm for 10 minutes at room temperature before injection (2%–3% of egg volume) into unfertilized eggs within 1 hour after spawning, prior to fertilization.

Injected embryos were cultured for 3 days in Millipore-filtered seawater (MFSW) at 18–20 °C. Metamorphosis of planula larvae into polyps was induced about 72 hours after fertilization by placing larvae (20–80/slide) on 75 × 50 mm glass slides in drops of 3–4 mL MFSW containing 1 μg/mL synthetic metamorphosis peptide (GNPPGLW-amide), followed by overnight incubation. Slides with fixed primary polyps were transferred to small aquariums kept at 24 °C, a temperature which favors the establishment of female colonies [68]. Primary polyps and young polyp colonies were fed twice a day with smashed *Artemia* nauplii until they were grown enough to be fed with swimming nauplii. Following colony vegetative expansion, a single well-growing colony on each slide was maintained as a founder. After several weeks of growth, polyp colonies were genotyped to assess mutation efficiency and mosaicism, and medusae were collected from the most strongly mutant colony (*MIHR* KO) for further experimentation.

### Genotyping

Genomic DNA from *Clytia* polyps was purified using DNeasy blood/tissue extraction kit (Qiagen). The *MIHR* target site was amplified by PCR using Phusion DNA polymerase. Primers used for genotyping are listed in S3 Table. PCR products were sequenced and mutation efficiency was assessed using TIDE analyses [69]. In cases in which Agarose gel analysis of the PCR product revealed large insertions or deletions, this was cloned into pGEM-T Easy vector and clones randomly selected for individual sequencing.

### Gonad spawning assays

Sexually mature *MIHR* mutant medusae from colonies n5-13 and n5-23 and wild-type medusae were cultured on the same day–night cycle. Individual gonads were dissected in the evening after afternoon spawning. To test the light response, one group of each was transferred to 100 μL MFSW in wells of 96-well plastic plates, covered overnight, and re-exposed to white light the following day. To test responses to MIH and cAMP, other groups of dissected gonads were cultured overnight in constant light, then transferred to 96-well plastic plates for two hours. MIH was added to wells as an equal volume of 200 nM WPRPamide (GenScript) stock in MFSW was added to give a final concentration of 100 nM. Alternatively, a stock of 20 mM bromo adenosine 3′,5′cyclic monophosphate (Br-cAMP–Fluka; Sigma Aldrich) in distilled water was added to give a final concentration of 4 mM. Gonads were washed after 5 minutes incubation in Br-cAMP. Oocyte maturation was scored after 30 minutes as germinal vesicle breakdown (GVBD), i.e., visible dissolution of the oocyte nuclear membrane upon entry into M phase, which in all cases was followed by spawning about 1 hour later. Gonads that showed premature maturation or spawning due to manipulation stress were excluded from analysis.

### Oocyte maturation assays

Fully grown oocytes were isolated manually from dissected gonads of wild-type and *MIHR* KO mutant jellyfish prepared as above. Maturation assays were performed in wells of 96-well plates or small plastic petri dishes lined with 2% agarose in MFSW. Spontaneous maturation occurs at a low frequency in control oocytes. To test MIH-induced maturation, 10 μL WPRPamide from 1 μM solution in MFSW was added to a final concentration of 100 nM. In some experiments, Br-cAMP from a 20 mM stock in H_2_O was used at a final concentration of 4 mM. GVBD was scored at 30 minutes, every 5 minutes, depending on experiments.

Antibody injection into isolated oocytes was performed using Nanoject or Narishige compressed air microinjection systems [70,71]. Solutions were centrifuged at 14,000 rpm at 4 °C for 5 minutes before use, and approximately 2% oocyte volume injected. An inhibitory anti-Gα_S_ antibody [33] was concentrated to 14 mg/mL in PBS by three passages through an ULTRAFREE spin column (Millipore- UFV5BQK25). A purified anti-GST antibody (Sigma 67781) at 8.3 mg/mL in PBS was used as a control.

### GPCR molecular phylogeny

Clustering analysis was performed using CLANS2 [63] with a BLOSUM62 matrix and a *p*-value cutoff of 1e-40 or 1e-50. Sequences were retrieved from NCBI (*Homo* and *Nematostella*) and from [28] for the deorphanized *Platynereis* GPCRs. *Nematostella* GPCRs were identified using HMMER [72]. Identifiers for the sequences used are given in S3 and S4 Texts. Multiple alignments were generated using Muscle [73] with default parameters. Positions containing more than 80% gaps were excluded from the alignment, provided in S5 Text. Phylogenetic analyses were performed using RaxML v8.2.9 [74] and the model PROTGAMMAGTR with Bootstrap support calculated from 500 replicates. The resulting tree file was visualized with FigTree (http://tree.bio.ed.ac.uk/software/figtree/).

### In situ hybridization

A urea-based protocol for in situ hybridization was used as previously [15,16,32].

### Immunofluorescence

For co-staining of neuropeptides and tyrosinated tubulin, dissected *Clytia* gonads and whole medusae were fixed overnight at 18 °C in HEM buffer (0.1 M HEPES, pH 6.9; 50 mM EGTA; 10 mM MgSO_4_) containing 3.7% formaldehyde, then washed five times in PBS containing 0.1% Tween20 (PBS-T). Treatment on ice with 50% methanol/PBS-T then 100% methanol plus storage in methanol at −20 °C improved visualization of microtubules in neural cells. Samples were rehydrated, washed several times in PBS-0.02% Triton X-100, then one time in PBS-0.2% Triton X-100 for 20 minutes, and again several times in PBS-0.02% Triton X-100. After overnight incubation at 4 °C in PBS with 3% BSA, they were incubated in a rabbit anti-PRPa antibody [15] and a rat monoclonal anti-Tyr tubulin (YL1/2, Thermo Fisher Scientific) in PBS/BSA at room temperature for 2 hours. After washes, the specimens were incubated with secondary antibodies (rhodamine goat anti-rabbit and Cy5 donkey anti-rat IgG; Jackson ImmunoResearch, West Grove, PA) overnight in PBS at 4 °C, and nuclei were stained using Hoechst dye 33258. Images were acquired using a Leica SP8 confocal microscope and maximum intensity projections of z-stacks prepared using ImageJ software. Phalloidin staining of fixed, non-methanol-treated gonads was performed as previously [16].

### Statistics

Fisher exact tests were performed at http://www.socscistatistics.com.

## Supporting information

S1 Fig Expression of *Clytia* GPCRs across tissues and life cycle stages. Heat map representing the expression of putative *Clytia* GPCRs in the different samples studied derived from RNA-seq data (see Methods), in which sequences are clustered according to similarity of their profiles across tissues and stages. Z-score values are color-coded to reflect significantly higher (dark blue) or lower (pale green) than average values—see z-value distribution in inset. Three main profile groups showed expression enriched in the oocytes (colored boxes). Numerical data available in S5 Data. EG, early gastrula; FGOo, fully grown oocyte; GEC, gonad ectoderm; GEN, gonad endoderm; GO, gonozooid; GPCR, G protein–coupled receptor; GrOo, growing oocyte; PH, polyp head; P2/3, 2/3-day old planula larva; RNA-seq, RNA sequencing; R1/R2, Biological Replicate1/2 for Illumina sequencing; St, stolon.
(JPG)

S2 Fig Phylogenetic tree file corresponding to Fig 6D, including identifiers for all sequences. Maximum likelihood analyses were performed using RaxML v8.2.9, and the model PROTGAMMAGTR with Bootstrap support was calculated from 500 replicates. Species color-coding as in Fig 6D: *Platynereis*, green; Human, blue, *Clytia*, orange; *Nematostella*, black.
(JPG)

S1 Text FASTA file of *Clytia* candidate GPCR sequences. GPCR, G protein–coupled receptor.
(DOC)

S2 Text FASTA file of the cloned *Clytia* candidate MIHR sequences, with their GenBank accession numbers in parentheses. MIHR, MIH receptor.
(DOC)

S3 Text Sequences used for CLANS2 clustering in Fig 6A and 6B. (DOC)

S4 Text Sequences used for CLANS2 clustering in Fig 6C. (DOC)

S5 Text FASTA file of the GPCR alignment used for phylogenetic analyses shown in Fig 6D. GPCR, G protein–coupled receptor.
(DOC)

S1 Table Details of PCR primers for GPCR cloning. GPCR, G protein–coupled receptor.
(XLSX)

S2 Table List of peptides used for GPCR deorphanization. GPCR, G protein–coupled receptor.
(XLSX)

S3 Table List of crRNA, CRISPR, and PCR primers for mutant genotyping. crRNA, CRISPR RNA.
(XLSX)

S1 Data Numerical data corresponding to Fig 1B. Raw luminescence values obtained in triplicate for each of the neuropeptide combinations tested against the MIHR in the deorphanization assay are provided. In the right-hand table, the raw luminescence values obtained in quadruple for the positive and negative controls are provided. In the lower table, means and standard errors of the mean of the luminescence values obtained for each neuropeptide combination and controls are also shown, as well as the values normalized to the positive control, which were used to prepare the graph shown in Fig 1B. MIHR, MIH receptor.
(XLSX)

S2 Data Numerical data corresponding to Fig 1C. The first three tabs show statistical values obtained using Prism 6 software (GraphPad, La Jolla, CA) from the raw luminescence values for 3 independent dose–response experiments. The first dataset (first tab) was used to create the graph shown in Fig 1C. The raw luminescence values are shown, as well as the values normalized with respect to the maximum. The 4th tab shows the different EC_50_ values calculated by Prism 6 software for each MIH neuropeptide from each of the 3 biological replicates, as well as the means shown in Fig 1C. MIH, maturation-inducing hormone.
(XLSX)

S3 Data Numerical data corresponding to Fig 4A and 4C. The first tab collects all the raw data from 3 independent gonad spawning assays. The means and standard deviations were used to prepare the graph shown in Fig 4A. Information corresponding to the Fisher exact tests is also shown. The second tab shows the raw data from 3 independent oocyte maturation assays. The means and standard deviations were used to prepare the graph in Fig 4C. Information corresponding to the Fisher exact tests is also shown.
(XLSX)

S4 Data Numerical data corresponding to Fig 5A. Data from 5 experiments are provided in the first tab. In experiments 1 and 2, oocytes were injected with either anti-Gα_s_ or PBS. In experiments 3–5, oocytes were injected with either anti-Gα_s_ or PBS or anti-GST. Injections were performed with a Narishige setup except for experiment 5, which used a nanoject apparatus and delivered less regular injections. Experiment 4 is illustrated in Fig 5A. The number of oocytes with intact GVs at the end of each successive treatment is indicated. The full details of these last three experiments are provided in tabs 2–4, including the timing and doses of MIH treatments, and the GV-intact oocyte counts at successive times. GST, Glutathione-S-Transferase; GV, Germinal Vesicle; MIH, maturation-inducing hormone.
(XLSX)

S5 Data Numerical data corresponding to S1 Fig. Normalized counts in the different sequenced samples for each GPCR candidate. GPCR, G protein–coupled receptor.
(XLSX)

## Abbreviations

7TM: 7 transmembrane domain
ASW: artificial seawater
Br-cAMP: bromoadenosine 3′,5′-cyclic monophosphate
cAMP: cyclic adenosine monophosphate
CHO-K1: Chinese Hamster ovary K1
FSH: follicle-stimulating hormone
GnIH: gonadotropin inhibitory hormone
GnRH: gonadotropin-releasing hormone
GPCR: G protein–coupled receptor
GST: Glutathione-S-Transferase
GVBD: germinal vesicle breakdown
HBSS: Hank’s balanced salt solution
KO: knockout
LH: luteinizing hormone
MFSW: Millipore-filtered seawater
MIH: maturation-inducing hormone
MIHR: MIH receptor
P2Y: purinoceptor
PBS-T: PBS containing 0.1% Tween20
PKA: protein kinase A/cAMP-dependent protein kinase
QRFP: pyroglutamylated RFamide peptide
RNA-seq: RNA sequencing
sgRNA: small guide RNA
TIDE: Tracking of Indels by Decomposition

## References

[1] Voronina E, Wessel GM. The regulation of oocyte maturation. Curr Top Dev Biol. 2003;58: 53–110. 10.1016/s0070-2153(03)58003-6 14711013

[2] Verlhac M-H, Terret M-E. Oocyte Maturation and Development. F1000Res. 2016;5 10.12688/f1000research.7892.1 26998245 PMC4786908

[3] Amiel A, Leclère L, Robert L, Chevalier S, Houliston E. Conserved functions for Mos in eumetazoan oocyte maturation revealed by studies in a cnidarian. Curr Biol. 2009;19: 305–311. 10.1016/j.cub.2008.12.054 19230670

[4] Von Stetina JR, Orr-Weaver TL. Developmental control of oocyte maturation and egg activation in metazoan models. Cold Spring Harb Perspect Biol. 2011;3: a005553 10.1101/cshperspect.a005553 21709181 PMC3179337

[5] Hartenstein V. The neuroendocrine system of invertebrates: a developmental and evolutionary perspective. J Endocrinol. 2006;190: 555–570. 10.1677/joe.1.06964 17003257

[6] Deguchi R, Takeda N, Stricker SA. Comparative biology of cAMP-induced germinal vesicle breakdown in marine invertebrate oocytes. Mol Reprod Dev. 2011;78: 708–725. 10.1002/mrd.21346 21774023

[7] Le Tissier P, Campos P, Lafont C, Romanò N, Hodson DJ, Mollard P. An updated view of hypothalamic-vascular-pituitary unit function and plasticity. Nat Rev Endocrinol. 2017;13: 257–267. 10.1038/nrendo.2016.193 27934864

[8] Kristiansen K. Molecular mechanisms of ligand binding, signaling, and regulation within the superfamily of G-protein-coupled receptors: molecular modeling and mutagenesis approaches to receptor structure and function. Pharmacol Ther. 2004;103: 21–80. 10.1016/j.pharmthera.2004.05.002 15251227

[9] Neves SR, Ram PT, Iyengar R. G protein pathways. Science. 2002;296: 1636–1639. 10.1126/science.1071550 12040175

[10] Oldham WM, Hamm HE. Heterotrimeric G protein activation by G-protein-coupled receptors. Nat Rev Mol Cell Biol. 2008;9: 60–71. 10.1038/nrm2299 18043707

[11] Chakravorty D, Assmann SM. G protein subunit phosphorylation as a regulatory mechanism in heterotrimeric G protein signaling in mammals, yeast, and plants. Biochem J. 2018;475: 3331–3357. 10.1042/BCJ20160819 30413679 PMC6347956

[12] Kalinowski RR, Berlot CH, Jones TLZ, Ross LF, Jaffe LA, Mehlmann LM. Maintenance of meiotic prophase arrest in vertebrate oocytes by a Gs protein-mediated pathway. Dev Biol. 2004;267: 1–13. 10.1016/j.ydbio.2003.11.011 14975713

[13] Freudzon L, Norris RP, Hand AR, Tanaka S, Saeki Y, Jones TLZ, et al Regulation of meiotic prophase arrest in mouse oocytes by GPR3, a constitutive activator of the Gs G protein. J Cell Biol. 2005;171: 255–265. 10.1083/jcb.200506194 16247026 PMC2171177

[14] Nader N, Dib M, Daalis A, Kulkarni RP, Machaca K. Role for endocytosis of a constitutively active GPCR (GPR185) in releasing vertebrate oocyte meiotic arrest. Dev Biol. 2014;395: 355–366. 10.1016/j.ydbio.2014.08.036 25220151

[15] Takeda N, Kon Y, Quiroga Artigas G, Lapébie P, Barreau C, Koizumi O, et al Identification of jellyfish neuropeptides that act directly as oocyte maturation-inducing hormones. Development. 2018;145 10.1242/dev.156786 29358214

[16] Quiroga Artigas G, Lapébie P, Leclère L, Takeda N, Deguchi R, Jékely G, et al A gonad-expressed opsin mediates light-induced spawning in the jellyfish. Elife. 2018;7 10.7554/eLife.29555 29303477 PMC5756024

[17] Takahashi T, Takeda N. Insight into the Molecular and Functional Diversity of Cnidarian Neuropeptides. International Journal of Molecular Sciences. 2015 pp. 2610–2625. 10.3390/ijms16022610 25625515 PMC4346854

[18] Bosch TCG, Klimovich A, Domazet-Lošo T, Gründer S, Holstein TW, Jékely G, et al Back to the Basics: Cnidarians Start to Fire. Trends Neurosci. 2017;40: 92–105. 10.1016/j.tins.2016.11.005 28041633 PMC5285349

[19] Grimmelikhuijzen CJP, Hauser F. Mini-review: the evolution of neuropeptide signaling. Regul Pept. 2012;177 Suppl: S6–9. 22726357 10.1016/j.regpep.2012.05.001

[20] Elphick MR, Mirabeau O, Larhammar D. Evolution of neuropeptide signalling systems. J Exp Biol. 2018;221 10.1242/jeb.151092 29440283 PMC5818035

[21] Freeman G, Ridgway EB. The role of cAMP in oocyte maturation and the role of the germinal vesicle contents in mediating maturation and subsequent developmental events in hydrozoans. Rouxs Arch Dev Biol. 1988;197: 197–211. 10.1007/BF02439427 28305628

[22] Takeda N, Kyozuka K, Deguchi R. Increase in intracellular cAMP is a prerequisite signal for initiation of physiological oocyte meiotic maturation in the hydrozoan *Cytaeis uchidae*. Dev Biol. 2006;298: 248–258. 10.1016/j.ydbio.2006.06.034 16884710

[23] Jékely G, Melzer S, Beets I, Kadow ICG, Koene J, Haddad S, et al The long and the short of it—a perspective on peptidergic regulation of circuits and behaviour. J Exp Biol. 2018;221 10.1242/jeb.166710 29439060

[24] Audet M, Bouvier M. Restructuring G-protein- coupled receptor activation. Cell. 2012;151: 14–23. 10.1016/j.cell.2012.09.003 23021212

[25] Jékely G. Global view of the evolution and diversity of metazoan neuropeptide signaling. Proc Natl Acad Sci U S A. 2013;110: 8702–8707. 10.1073/pnas.1221833110 23637342 PMC3666674

[26] Leclère L, Horin C, Chevalier S, Lapébie P, Dru P, Peron S, et al The genome of the jellyfish *Clytia hemisphaerica* and the evolution of the cnidarian life-cycle. Nat Ecol Evol. 2019;3: 801–810. 10.1038/s41559-019-0833-2 30858591

[27] Tunaru S, Lättig J, Kero J, Krause G, Offermanns S. Characterization of Determinants of Ligand Binding to the Nicotinic Acid Receptor GPR109A (HM74A/PUMA-G). Molecular Pharmacology. 2005 pp. 1271–1280. 10.1124/mol.105.015750 16099840

[28] Bauknecht P, Jékely G. Large-Scale Combinatorial Deorphanization of *Platynereis* Neuropeptide GPCRs. Cell Rep. 2015;12: 684–693. 10.1016/j.celrep.2015.06.052 26190115

[29] Nielsen SKD, Koch TL, Hauser F, Garm A, Grimmelikhuijzen CJP. De novo transcriptome assembly of the cubomedusa *Tripedalia cystophora*, including the analysis of a set of genes involved in peptidergic neurotransmission. BMC Genomics. 2019;20: 175 10.1186/s12864-019-5514-7 30836949 PMC6402141

[30] Hayakawa E, Watanabe H, Menschaert G, Holstein TW, Baggerman G, Schoofs L. A combined strategy of neuropeptide prediction and tandem mass spectrometry identifies evolutionarily conserved ancient neuropeptides in the sea anemone *Nematostella vectensis*. PLoS ONE. 2019;14: e0215185 10.1371/journal.pone.0215185 31545805 PMC6756747

[31] Condamine T, Jager M, Leclère L, Blugeon C, Lemoine S, Copley RR, et al Molecular characterisation of a cellular conveyor belt in *Clytia* medusae. Dev Biol. 2019; 10.1016/j.ydbio.2019.09.001 31509769

[32] Sinigaglia C, Thiel D, Hejnol A, Houliston E, Leclère L. A safer, urea-based in situ hybridization method improves detection of gene expression in diverse animal species. Dev Biol. 2018;434: 15–23. 10.1016/j.ydbio.2017.11.015 29197505

[33] Gallo CJ, Hand AR, Jones TL, Jaffe LA. Stimulation of *Xenopus* oocyte maturation by inhibition of the G-protein alpha S subunit, a component of the plasma membrane and yolk platelet membranes. J Cell Biol. 1995;130: 275–284. 10.1083/jcb.130.2.275 7615631 PMC2199928

[34] Mehlmann LM, Jones TLZ, Jaffe LA. Meiotic arrest in the mouse follicle maintained by a Gs protein in the oocyte. Science. 2002;297: 1343–1345. 10.1126/science.1073978 12193786

[35] Mirabeau O, Joly J-S. Molecular evolution of peptidergic signaling systems in bilaterians. Proc Natl Acad Sci U S A. 2013;110: E2028–37. 10.1073/pnas.1219956110 23671109 PMC3670399

[36] Zandawala M, Moghul I, Yañez Guerra LA, Delroisse J, Abylkassimova N, Hugall AF, et al Discovery of novel representatives of bilaterian neuropeptide families and reconstruction of neuropeptide precursor evolution in ophiuroid echinoderms. Open Biol. 2017;7 10.1098/rsob.170129 28878039 PMC5627052

[37] Thiel D, Franz-Wachtel M, Aguilera F, Hejnol A. Xenacoelomorph Neuropeptidomes Reveal a Major Expansion of Neuropeptide Systems during Early Bilaterian Evolution. Molecular Biology and Evolution. 2018 pp. 2528–2543. 10.1093/molbev/msy160

[38] Anctil M. Chemical transmission in the sea anemone *Nematostella vectensis*: A genomic perspective. Comp Biochem Physiol Part D Genomics Proteomics. 2009;4: 268–289. 10.1016/j.cbd.2009.07.001 20403752

[39] Haccard O, Jessus C. Oocyte Maturation, Mos and Cyclins—A Matter of Synthesis: Two Functionally Redundant Ways to Induce Meiotic Maturation. Cell Cycle. 2006 pp. 1152–1159. 10.4161/cc.5.11.2800 16760654

[40] Nagahama Y, Yamashita M. Regulation of oocyte maturation in fish. Dev Growth Differ. 2008;50 Suppl 1: S195–219. 18482399 10.1111/j.1440-169X.2008.01019.x

[41] Kishimoto T. MPF-based meiotic cell cycle control: Half a century of lessons from starfish oocytes. Proceedings of the Japan Academy, Series B. 2018 pp. 180–203. 10.2183/pjab.94.013 29643273 PMC5968197

[42] Jaffe LA, Egbert JR. Regulation of Mammalian Oocyte Meiosis by Intercellular Communication Within the Ovarian Follicle. Annual Review of Physiology. 2017 pp. 237–260. 10.1146/annurev-physiol-022516-034102 27860834 PMC5305431

[43] Lutz LB, Kim B, Jahani D, Hammes SR. G protein beta gamma subunits inhibit nongenomic progesterone-induced signaling and maturation in *Xenopus laevis* oocytes. Evidence for a release of inhibition mechanism for cell cycle progression. J Biol Chem. 2000;275: 41512–41520. 10.1074/jbc.M006757200 11018039

[44] Zhu Y, Rice CD, Pang Y, Pace M, Thomas P. Cloning, expression, and characterization of a membrane progestin receptor and evidence it is an intermediary in meiotic maturation of fish oocytes. Proc Natl Acad Sci U S A. 2003;100: 2231–2236. 10.1073/pnas.0336132100 12574519 PMC151323

[45] Ben-Yehoshua LJ, Lewellyn AL, Thomas P, Maller JL. The Role of *Xenopus* Membrane Progesterone Receptor β in Mediating the Effect of Progesterone on Oocyte Maturation. Molecular Endocrinology. 2007 pp. 664–673. 10.1210/me.2006-0256 17185392

[46] Nader N, Courjaret R, Dib M, Kulkarni RP, Machaca K. Release from *Xenopus* oocyte prophase I meiotic arrest is independent of a decrease in cAMP levels or PKA activity. Development. 2016;143: 1926–1936. 10.1242/dev.136168 27122173

[47] Patel SR, Murphy KG, Thompson EL, Patterson M, Curtis AE, Ghatei MA, et al Pyroglutamylated RFamide Peptide 43 Stimulates the Hypothalamic-Pituitary-Gonadal Axis via Gonadotropin-Releasing Hormone in Rats. Endocrinology. 2008 pp. 4747–4754. 10.1210/en.2007-1562 18535111

[48] Tsutsui K, Bentley GE, Kriegsfeld LJ, Osugi T, Seong JY, Vaudry H. Discovery and Evolutionary History of GnIH and Kisspeptin: New Key Neuropeptides Controlling Reproduction. Journal of Neuroendocrinology. 2010 p. no–no. 10.1111/j.1365-2826.2010.02018.x 20456604 PMC2909878

[49] Hu G, Lin C, He M, Wong AOL. Neurokinin B and reproductive functions: “KNDy neuron” model in mammals and the emerging story in fish. Gen Comp Endocrinol. 2014;208: 94–108. 10.1016/j.ygcen.2014.08.009 25172151

[50] Saberi A, Jamal A, Beets I, Schoofs L, Newmark PA. GPCRs Direct Germline Development and Somatic Gonad Function in Planarians. PLoS Biol. 2016;14: e1002457 10.1371/journal.pbio.1002457 27163480 PMC4862687

[51] Ohno H, Yoshida M, Sato T, Kato J, Miyazato M, Kojima M, et al Luqin-like RYamide peptides regulate food-evoked responses in *C*. *elegans*. eLife 2017;6: e28877 10.7554/eLife.28877 28847365 PMC5576490

[52] Gomes I, Bobeck EN, Margolis EB, Gupta A, Sierra S, Fakira AK, et al Identification of GPR83 as the receptor for the neuroendocrine peptide PEN. Sci Signal. 2016;9: ra43. 10.1126/scisignal.aad0694 PMC5147544 27117253

[53] Chartrel N, Picot M, El Medhi M, Arabo A, Berrahmoune H, Alexandre D, et al The Neuropeptide 26RFa (QRFP) and Its Role in the Regulation of Energy Homeostasis: A Mini-Review. Front Neurosci. 2016;10: 549 10.3389/fnins.2016.00549 27965532 PMC5126098

[54] Navarro VM, Fernández-Fernández R, Nogueiras R, Vigo E, Tovar S, Chartrel N, et al Novel role of 26RFa, a hypothalamic RFamide orexigenic peptide, as putative regulator of the gonadotropic axis. J Physiol. 2006;573: 237–249. 10.1113/jphysiol.2006.106856 16543265 PMC1779712

[55] Wójcik-Gładysz A, Polkowska J. Neuropeptide Y—a neuromodulatory link between nutrition and reproduction at the central nervous system level. Reprod Biol. 2006;6 Suppl 2: 21–28. 17220938

[56] Tsutsui K, Ubuka T. GnIH Control of Feeding and Reproductive Behaviors. Front Endocrinol. 2016;7: 170. 10.3389/fendo.2016.00170 PMC5186799 28082949

[57] Al-Anzi B, Armand E, Nagamei P, Olszewski M, Sapin V, Waters C, et al The leucokinin pathway and its neurons regulate meal size in *Drosophila*. Curr Biol. 2010;20: 969–978. 10.1016/j.cub.2010.04.039 20493701 PMC2896026

[58] Maeda T., Nakamura Y., Shiotani H., Hojo M.K., Yoshii T., Ida T., et al Suppressive effects of dRYamides on feeding behavior of the blowfly, *Phormia regina*. Zoological Lett. 2015;1: 35 10.1186/s40851-015-0034-z 26649188 PMC4672552

[59] Shahjahan M, Kitahashi T, Parhar IS. Central pathways integrating metabolism and reproduction in teleosts. Front Endocrinol. 2014;5: 36. 10.3389/fendo.2014.00036 PMC3971181 24723910

[60] Lapébie P, Ruggiero A, Barreau C, Chevalier S, Chang P, Dru P, et al Differential responses to Wnt and PCP disruption predict expression and developmental function of conserved and novel genes in a cnidarian. PLoS Genet. 2014;10: e1004590 10.1371/journal.pgen.1004590 25233086 PMC4169000

[61] Fu L, Niu B, Zhu Z, Wu S, Li W. CD-HIT: accelerated for clustering the next-generation sequencing data. Bioinformatics. 2012;28: 3150–3152. 10.1093/bioinformatics/bts565 23060610 PMC3516142

[62] Langmead B, Salzberg SL. Fast gapped-read alignment with Bowtie 2. Nat Methods. 2012;9: 357–359. 10.1038/nmeth.1923 22388286 PMC3322381

[63] Frickey T, Lupas A. CLANS: a Java application for visualizing protein families based on pairwise similarity. Bioinformatics. 2004;20: 3702–3704. 10.1093/bioinformatics/bth444 15284097

[64] Gibson DG, Young L, Chuang R-Y, Craig Venter J, Hutchison CA, Smith HO. Enzymatic assembly of DNA molecules up to several hundred kilobases. Nature Methods. 2009 pp. 343–345. 19363495 10.1038/nmeth.1318

[65] Offermanns S, Simon MI. G alpha 15 and G alpha 16 couple a wide variety of receptors to phospholipase C. J Biol Chem. 1995;270: 15175–15180. 10.1074/jbc.270.25.15175 7797501

[66] Baubet V, Le Mouellic H, Campbell AK, Lucas-Meunier E, Fossier P, Brúlet P. Chimeric green fluorescent protein-aequorin as bioluminescent Ca2+ reporters at the single-cell level. Proc Natl Acad Sci U S A. 2000;97: 7260–7265. 10.1073/pnas.97.13.7260 10860991 PMC16533

[67] Momose T, De Cian A, Shiba K, Inaba K, Giovannangeli C, Concordet J-P. High doses of CRISPR/Cas9 ribonucleoprotein efficiently induce gene knockout with low mosaicism in the hydrozoan *Clytia hemisphaerica* through microhomology-mediated deletion. Sci Rep. 2018;8: 11734 10.1038/s41598-018-30188-0 30082705 PMC6078951

[68] Carré D, Carré C. Origin of germ cells, sex determination, and sex inversion in medusae of the genus *Clytia* (Hydrozoa, leptomedusae): the influence of temperature. J Exp Zool. 2000;287: 233–242. 10.1002/1097-010x(20000801)287:3<233::aid-jez5>3.3.co;2-6 10900443

[69] Brinkman EK, Chen T, Amendola M, van Steensel B. Easy quantitative assessment of genome editing by sequence trace decomposition. *Nucleic Acids Res*. 2014;42(22):e168 10.1093/nar/gku936 25300484 PMC4267669

[70] Momose T, Houliston E. Two oppositely localised frizzled RNAs as axis determinants in a cnidarian embryo. PLoS Biol. 2007;5: e70 10.1371/journal.pbio.0050070 17355179 PMC1820609

[71] Yasuo H, McDougall A. Practical Guide for Ascidian Microinjection: *Phallusia mammillata*. Adv Exp Med Biol. 2018;1029: 15–24. 10.1007/978-981-10-7545-2_3 29542077

[72] Potter SC, Luciani A, Eddy SR, Park Y, Lopez R, Finn RD. HMMER web server: 2018 update. Nucleic Acids Res. 2018;46: W200–W204. 10.1093/nar/gky448 29905871 PMC6030962

[73] Edgar RC. MUSCLE: a multiple sequence alignment method with reduced time and space complexity. BMC Bioinformatics. 2004;5: 113 10.1186/1471-2105-5-113 15318951 PMC517706

[74] Stamatakis A. RAxML-VI-HPC: maximum likelihood-based phylogenetic analyses with thousands of taxa and mixed models. Bioinformatics. 2006 pp. 2688–2690. 10.1093/bioinformatics/btl446 16928733

