# Supplementary figures and images for: A G protein–coupled receptor mediates neuropeptide-induced oocyte maturation in the jellyfish Clytia
Source: PLoS Biol. 2020 Mar 3;18(3):e3000614. doi: 10.1371/journal.pbio.3000614 (PMC7053711; doi:10.1371/journal.pbio.3000614)

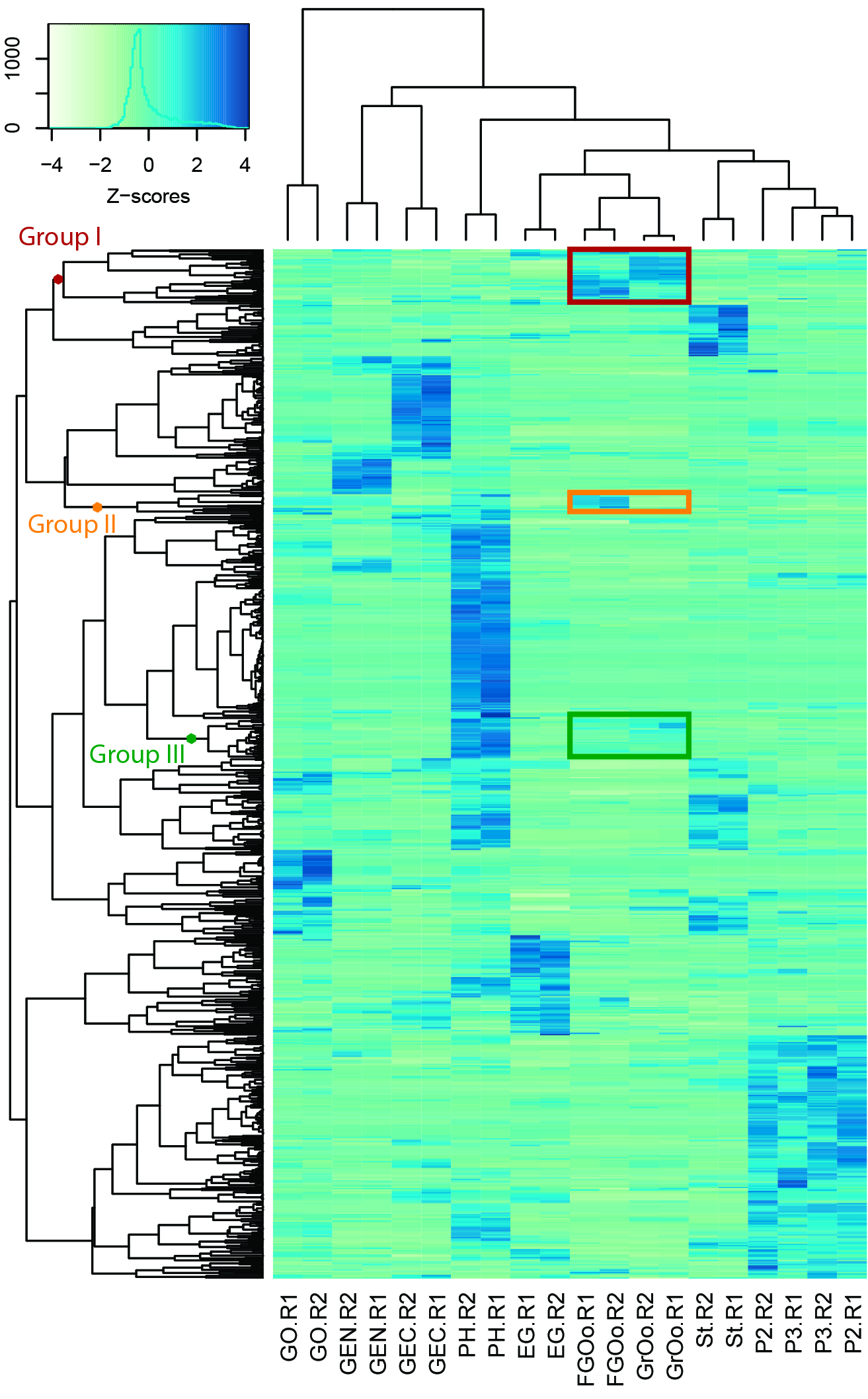

Supplement: S1 Fig — Heat map representing the expression of putative Clytia GPCRs in the different samples studied derived from RNA-seq data (see Methods), in which sequences are clustered according to similarity of their profiles across tissues and stages. Z-score values are color-coded to reflect significantly higher (dark blue) or lower (pale green) than average values—see z-value distribution in inset. Three main profile groups showed expression enriched in the oocytes (colored boxes). Numerical data available in S5 Data. EG, early gastrula; FGOo, fully grown oocyte; GEC, gonad ectoderm; GEN, gonad endoderm; GO, gonozooid; GPCR, G protein–coupled receptor; GrOo, growing oocyte; PH, polyp head; P2/3, 2/3-day old planula larva; RNA-seq, RNA sequencing; R1/R2, Biological Replicate1/2 for Illumina sequencing; St, stolon. (JPG) [file pbio.3000614.s001.jpg]

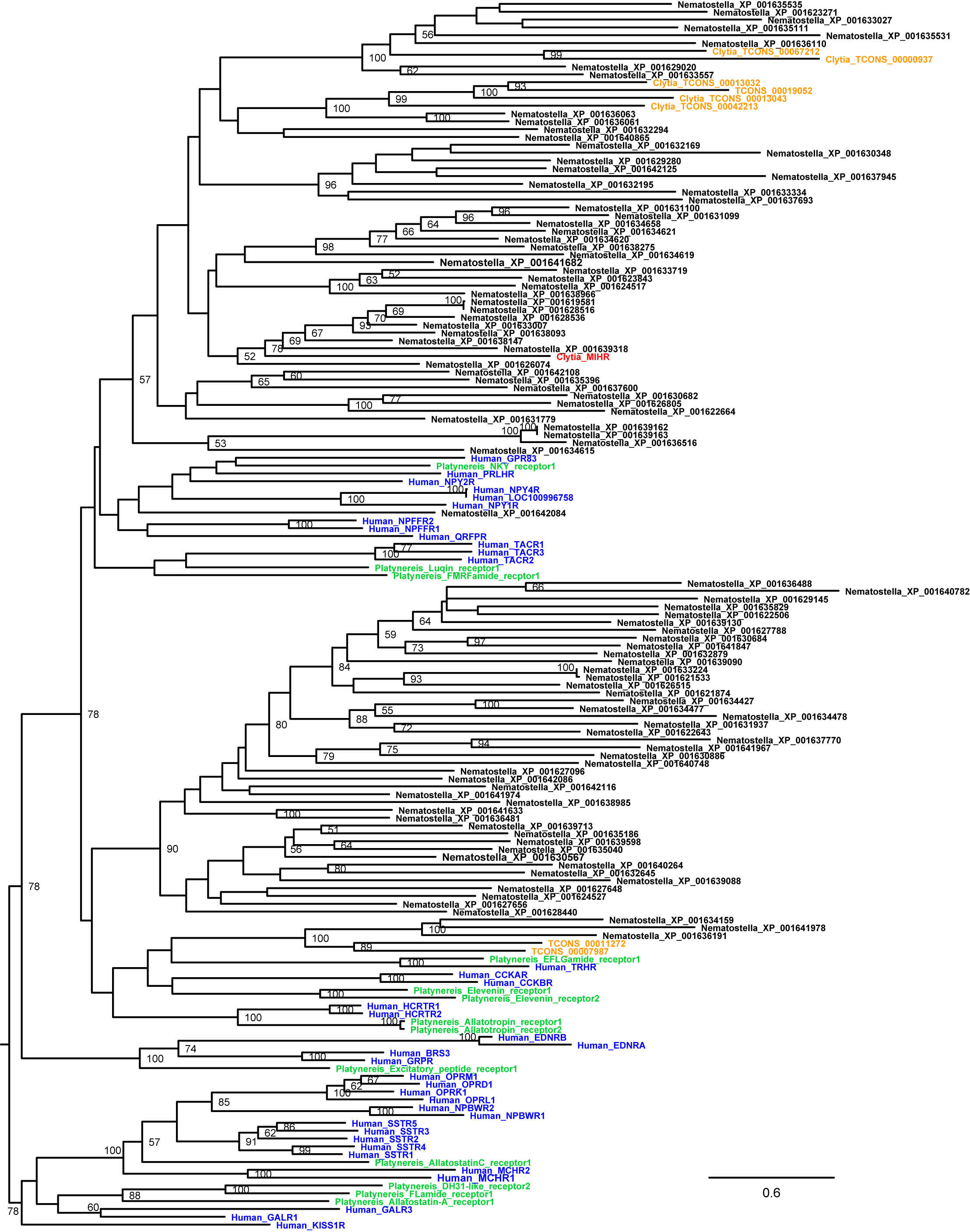

Supplement: S2 Fig — Maximum likelihood analyses were performed using RaxML v8.2.9, and the model PROTGAMMAGTR with Bootstrap support was calculated from 500 replicates. Species color-coding as in Fig 6D: Platynereis, green; Human, blue, Clytia, orange; Nematostella, black. (JPG) [file pbio.3000614.s002.jpg]
